# Preparation and biological assessment of some aromatic hydrazones derived from hydrazides of phenolic acids and aromatic aldehydes

**DOI:** 10.1016/j.heliyon.2020.e05019

**Published:** 2020-09-29

**Authors:** Ziad Moussa, Mohammed Al-Mamary, Sultan Al-Juhani, Saleh A. Ahmed

**Affiliations:** a Department of Chemistry, College of Science, United Arab Emirates University, P.O. Box 15551, Al Ain, United Arab Emirates; b Chemistry Department, Faculty of Science, Taibah University, PO Box 30002, Code, 14177, Al Madinah Al Almunawarrah, Saudi Arabia; c Chemistry Department of Chemistry, Faculty of Applied Science, Umm Al-Qura University, 21955 Makkah, Saudi Arabia; d Department of Chemistry, Faculty of Science, Assiut University, 71516 Assiut, Egypt

**Keywords:** Organic chemistry, Aromatic hydrazones, Antioxidant activity, Cholinesterase inhibition, Tyrosinase inhibition, DPPH, ABTS, FTC

## Abstract

There has been substantial interest over the past many years in the design of novel chemical compounds containing the azomethine group (-NH-N=CH) and exhibiting various medicinal properties such as antibacterial, antiviral, antifungal, and anti-inflammatory activities. Herein, hydrazones were synthesized via the chemical reaction of substituted aromatic hydrazides with various aromatic aldehydes. The obtained products were confirmed using different physical and spectroscopic techniques, such as m.p., IR, ^1^H-NMR and ^13^C-NMR. The present study was designed to synthesize different aromatic hydrazones assembled by various combinations of aromatic hydrazides and aromatic benzaldehydes containing different substituents such as hydroxyl and polyhydroxyl groups as key structural features. Thus, incorporating such moieties and simultaneously creating highly-conjugated systems was expected to create novel species to mimic as much as possible natural phenolics, chalcones and stilbenes. Compounds of aromatic hydrazones synthesized in the present study were tested *in vitro* for their direct and indirect antioxidant activities using different methods such as DPPH, ABTS and FTC. The antioxidant activities of the new compounds ranged from very weak to very high activity. In addition, the inhibition of tyrosinase and cholinesterase by these compounds was tested. The new compounds containing two or three hydroxyl groups attached to aldehyde rings exhibited significantly greater inhibition effects on tyrosinase or cholinesterase activities in comparison to other compounds of the same series containing only one hydroxyl group.

## 1 Introduction

The hydrazone function is a privileged moiety and plays a noteworthy role in the area of medicinal chemistry. Consequently, the chemotherapeutic potential of this class of organic compounds provided the impetus that attracted many researchers involved in drug discovery and development to explore the synthesis and testing of their biological activity in the hope of finding some hydrazones with potent bioactivities. Aromatic hydrazones are compounds derived from phenolic acid hydrazides and aromatic aldehydes. Generally, these compounds contain the azomethine group (-NH-N=CH-) and are prepared by refluxing stoichiometric amounts of the aromatic hydrazide and aldehyde or ketone dissolved in a suitable solvent. Therefore, hydrazides and hydrazones are nowadays of considerable technical and commercial importance due to their wide utilization as drugs in medicine and as versatile ligands in coordination chemistry (Hueso-Urena et al., 1999; Özdemir and Gülçin, 2008). It is noted that the growing interest in the chemistry of hydrazones is related to their wide spectrum of bioactivities.

Many organic compounds containing the azomethine group (-NH-N=CH-) have been synthesized and tested for their biological activities, proving very effective as antimicrobial (Anbazhagan and Sankaran, 2015; Kaki et al., 2014; Narisetty et al., 2013; Pieczonka et al., 2013; Moldovan et al., 2011; Popiołek and Biernasiuk, 2016, 2017; Rambabu et al., 2015; Rasras et al., 2010; Rutkauskas et al., 2013; Satyanarayana et al., 2014; Kumar et al., 2015) and anticancer agents (Terzioglu and Gürsoy, 2003). Others have synthesized organic compounds containing the hydrazone moiety and reported such species to exhibit antibacterial activities when tested against different bacterial strains (Hamdi et al., 2011; Govindasami et al., 2011; Wu et al., 2012a, Wu et al., 2012b; Backes et al., 2014; Dai et al., 2015; Wang et al., 2016; Zhang et al., 2014; Wu et al., 2012a, Wu et al., 2012b). Various hydrazone derivatives have been synthesized and shown to be effective antifungal agents (Backes et al., 2014). Abdel-Aal and co-workers (2008) prepared different hydrazone derivatives that were tested against hepatitis-A virus and herpes simplex virus-1. Some of these chemical agents exhibited high antiviral activity against the African green monkey (AGM-27) strain of hepatitis A virus (HAV-27) and herpes simplex virus-1. On the other hand, Galayko et al. (2010) studied some hydrazone compounds which demonstrated high prophylactic activity against vesicular stomatitis virus (VSV) and repressed viral replication in primarily infested cells. Recently some hydrazone derivatives were synthesized and tested *in vitro* for their cytotoxicity and anticancer activity. These new reagents have been tested against different human tumor cell lines and have shown to be promising anti-cancer agents (Kaplánek et al., 2015; Sundaree et al., 2016).

Recently, hydrazones have been investigated for their ability to remove free radicals, since the latter species have been identified as main culprits in different disease states arising from oxidative stress. These include skin cancer, Alzheimer's disease (AD), cardiovascular diseases, inflammation, among others. For instance, Reis recently reported novel hydrazone hybrid derivatives that exhibited antioxidant and photoprotective activities and proved useful for the prevention of skin cancer and use in sunscreen formulations (Reis et al., 2014). These particular compounds were constructed via the molecular hybridization of t-resveratrol, avobenzone and octyl methoxycinnamate. Four of the reported compounds showed Sun Protector Factor (SPF) exceeding that of t-resveratrol and are considered efficient broad-spectrum UVA and UVA filters. As for species that act as multifunctional agents targeting Alzheimer's disease, several hydrazide based Schiff bases were recently described by Rahim (Rahim et al., 2016) and were shown to inhibit acetylcholinesterase and butyrylcholinesterase, albeit to various degrees, when compared to the standard physostigmine. The inhibition of xanthine oxidase (XO) by hydrazones derived from hydroxy-substituted benzaldehydes has also been reported (Leigh et al., 2011). XO inhibitors are prospective agents for treatment of chronic heart failure and cardiovascular disease. The anti-inflammatory effect of derivatives of *N*-arylidene hydrazones has also been investigated *in vitro* as well as in inhibiting the activities of the proteolytic enzymes cathepsin Е and human neutrophil elastase which play important role in the development of autoimmune diseases (Nurkenov et al., 2017).

Therefore, different types of organic compounds containing the azomethine moiety have been previously synthesized and tested for their ability to remove free radicals (Taha et al., 2013; Kareem et al., 2015; Khaledi et al., 2011). The anti-depressant activity of hydrazones have also been evaluated by several researchers who synthesized and evaluated the effects of hydrazone derivatives on depression (Ergenç and Gunay, 1998; de Oliveiraa et al., 2011; Can et al., 2012, 2017). Other reported biological properties of synthesized organic compounds containing the hydrazone moiety include antiplatelet (Fraga et al., 2000; Silva et al., 2004), analgesic (Bhandari et al., 2008; Lima et al., 2000), anti-inflammatory (Lima et al., 2000; Salgın-Gökşen et al., 2007; Mohamed Eissa et al., 2012), and antimalarial activities (Walcourt et al., 2004; Gemma et al., 2006; Melnyk et al., 2006).

Due to the considerable attention that has been given in recent years to the chemistry of hydrazones and polyphenols and the diverse biological and pharmaceutical applications encompassed by such a class, we were encouraged to delve further into this area of research. Therefore, the present study was designed to construct structurally-different hydrazones derived from the coupling of benzoic and phenolic acids hydrazides and aromatic aldehydes containing different substituents, most notably the hydroxyl groups. These compounds feature highly-conjugated systems enhanced by the presence of hydroxyl groups. Thus, it was expected that the new compounds would closely mimic natural chalcones and stilbenes, which are well known natural antioxidants. There is continuous need for new chemical entities, such as antioxidants, that can have desired biological attributes with minimal or no side effects, and compounds to treat and prevent diseases related to oxidative stress, such as, skin cancer, AD and others.

## 2 Materials and methods

### 2.1 Material

#### 2.1.1 Reagents

Benzoic acid, phenolic acids, aromatic aldehydes, hydrazine solution (78–82%), DPPH, ABTS, L-DOPA, and acetylthiocholine iodide were obtained from Sigma and used without any further purification. The cholinesterase enzyme was purchased from Sigma, while tyrosinase was prepared in our laboratory from mushroom. Methyl esters of benzoic and phenolic acids and also benzoic and phenolic acids hydrazides were prepared in our laboratory according to published procedures.

#### 2.1.2 Equipment

The chemical structures of the phenolic acids hydrazones were confirmed using various instruments and techniques, such as, melting point instrument (model SMP10, Barloworld Scientific), IR spectrophotometer (FT IR-8400S, Shimadzu), NMR spectrophotometer (^1^H-NMR, ^13^C-NMR, and ^13^C-DEPT 90 NMR, Bruker Avance II 400 MHz), and UV/VIS spectrophotometer (Thermo Scientific, Genesys 10S UV-VIS Spectrophotometer). The obtained data was consistent with those reported in the literature for all known compounds.

### 2.2 Methods

#### 2.2.1 General synthesis of benzoic and phenolic acids hydrazones (**1**–**21**)

The aromatic aldehyde (10 mmol) was dissolved in ethanol and was treated with a solution of benzoic acid hydrazide (10 mmol) or phenolic acid hydrazide (10 mmol) in ethanol (25 mL), followed by three drops of glacial acetic acid. The mixture was stirred vigorously and refluxed using a water bath for three hours. The reaction mixture was then cooled down to ambient temperature and the resulting precipitate was filtered off by gravity filtration, dried and recrystallized from ethanol to give products **1**–**21**

(Scheme 1).

**Figure:**
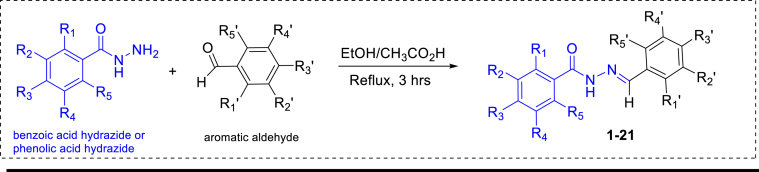

**Scheme 1:**
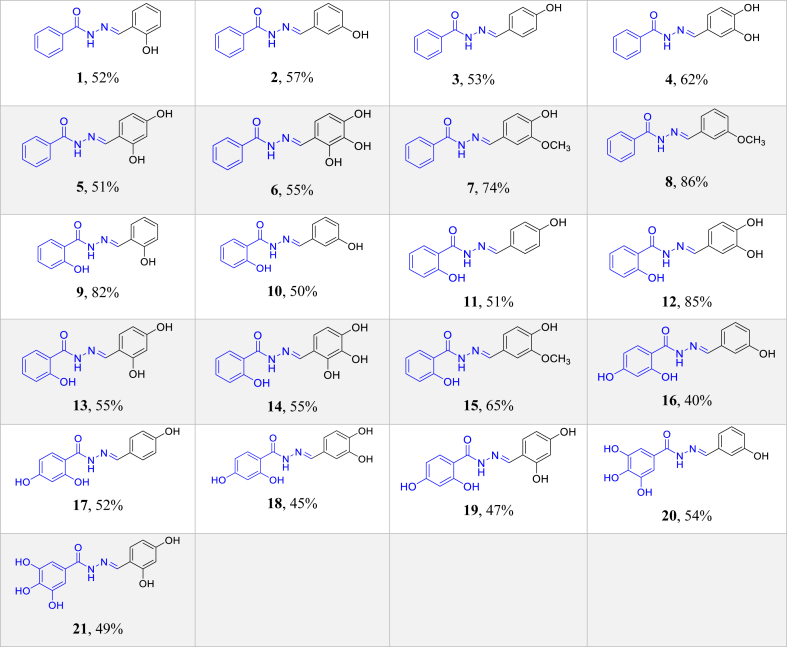
Synthesis of benzoic and phenolic acids hydrazones.

#### 2.2.2 Experimental data

The yields, m.p. and spectral data of compounds (**1**–**21**) are presented below and the corresponding spectra are shown in the supporting information section:

**(E)-N'-(2-hydroxybenzylidene)benzohydrazide** (**1**) (Edward et al., 1988):- **Yield (%):** 52%; **m.p. °C:** 175–177; **IR:** 3267 (N-H & OH), 3061 (=C-H), 1666 (C=O), 1645 (C=N), 1543-1483 (C=C Ar ring), 1360, 1263 (C-O); ^**1**^**H-NMR** (1:1 DMSO-d_6_/CDCl_3_, 400 MHz): δ 8.50 (s, 1H, C**H** = N), 7.90 (d, *J* = 8.0 Hz, 2H), 7.54–7.48 (m, 1H), 7.47–7.40 (m, 2H), 7.27–7.18 (m, 2H), 6.90 (d, *J* = 8.0 Hz, 1H), 7.84 (t, *J* = 8.0 Hz, 1H); ^**13**^**C-NMR** (1:1 DMSO-d_6_/CDCl_3_, 100 MHz): δ 163.1 (C=O), 157.9 (C-O), 149.5 (N=CH), 132.6 (Ar**C**-C=O), 131.4 (CH), 130.8 (CH), 130.1 (2xCH), 127.9 (2xCH), 127.3 (CH), 118.6 (CH), 117.6 (Ar**C**CH=N), 116.3 (CH) ppm.

**(E)-N'-(3-hydroxybenzylidene)benzohydrazide** (**2**) (Kim et al., 2016):- **Yield (%):** 57%; **m.p. °C:** 209–211; **IR:** 3250 (N-H & OH), 3066 (=C-H), 1638 (C=O), 1610 (C=N), 1577-1533 (C=C Ar ring), 1281 (C-O); ^**1**^**H-NMR** (1:1 DMSO-d_6_/CDCl_3_, 400 MHz): δ 11.50 (s,1H, NH), 9.09 (s,1H, OH), 8.30 (s, 1H, C**H** = N), 7.86 (d, *J* = 8.0 Hz, 2H), 7.50–7.42 (m, 1H), 7.41–7.45 (m, 2H), 7.26–7.17 (m, 1H), 7.16–7.05 (m, 2H), 6.79 (d, *J* = 8.0 Hz, 1H); ^**13**^**C-NMR** (1:1 DMSO-d_6_/CDCl_3_, 100 MHz): δ 163.8 (C=O), 157.2 (C-O), 148.2 (N=CH), 135.1 (Ar**C**-C=O), 133.3 (Ar**C**CH=N), 131.1 (CH), 129.1 (CH), 127.9 (2xCH), 127.3 (2xCH), 118.6 (CH), 117.2 (CH), 113.2 (CH) ppm.

**(E)-N'-(4-hydroxybenzylidene)benzohydrazide** (**3**) (Kaupp et al., 2000):- **Yield (%):** 53%; **m.p. °C:** 242–244; **IR:** 3262 (N-H & OH), 3073 (=C-H), 1647 (C=O), 1600 (C=N), 1499 (C=C Ar ring), 1280 (C-O); ^**1**^**H-NMR** (1:1 DMSO-d_6_/CDCl_3_, 400 MHz): δ 11.33 (s,1H, NH), 9.39 (s,1H, OH), 8.27 (s,1H, C**H** = N), 7.84 (d, *J* = 8.0 Hz, 2H), 7.52 (d, *J* = 8.0 Hz, 2H), 7.49–7.42 (m, 1H), 7.41–7.35 (m, 2H), 6.76 (d, *J* = 8.0 Hz, 2H); ^**13**^**C-NMR** (1:1 DMSO-d_6_/CDCl_3_, 100 MHz): δ 163.6 (C=O), 159.1 (C-O), 148.4 (N=CH), 133.5 (Ar**C**-C=O), 130.9 (CH), 128.7 (2xCH), 127.8 (2xCH), 127.2 (2xCH), 124.9 (Ar**C**CH=N), 115.2 (2xCH) ppm.

**(E)-N'-(3,4-dihydroxybenzylidene)benzohydrazide** (**4**) (Oliveira et al., 2014):- **Yield (%):** 62%; **m.p. °C:** 220–222; **IR:** 3262 (N-H & OH), 3058 (=C-H), 1646 (C=O), 1600 (C=N), 1506 (C=C Ar ring), 1280 (C-O); ^**1**^**H-NMR** (1:1 DMSO-d_6_/CDCl_3_, 400 MHz): δ 11.28 (s,1H, NH), 8.63 (s,1H, OH), 8.19 (s,1H, C**H** = N), 7.83 (d, *J* = 8.0 Hz, 2H), 7.54–7.34 (m, 3H), 7.30 (s,1H), 6.93 (d, *J* = 8.0 Hz, 1H), 6.74 (d, *J* = 8.0 Hz, 1H); ^**13**^**C-NMR** (1:1 DMSO-d_6_/CDCl_3_, 100 MHz): δ 163.6 (C=O), 148.5 (N=CH), 147.3 (C-O), 144.9 (C-O), 133.5 (Ar**C**-C=O), 130.9 (CH), 127.8 (2xCH), 127.2 (2xCH), 125.7 (Ar**C**CH=N), 120.4 (CH), 114.9 (CH), 113.1 (CH) ppm.

**(E)-N'-(2,4-dihydroxybenzylidene)benzohydrazide** (**5**) (Melnyk et al., 2006):- **Yield (%):** 51%; **m.p. °C:** 268–270; **IR:** 3246 (N-H & OH), 3065 (=C-H), 1647 (C=O), 1600 (C=N), 1506 (C=C Ar ring), 1271 (C-O); ^**1**^**H-NMR** (1:1 DMSO-d_6_/CDCl_3_, 400 MHz): δ 11.58 (s,1H, NH), 9.47 (s,1H, OH), 8.37 (s,1H, C**H** = N), 7.85 (d, *J* = 8.0 Hz, 2H), 7.51–7.33 (m, 3H), 6.99 (d, *J* = 8.0 Hz, 1H), 6.32 (d, *J* = 8.0 Hz, 2H); ^**13**^**C-NMR** (1:1 DMSO-d_6_/CDCl_3_, 100 MHz): δ 163.0 (C=O), 160.5 (C-O), 159.9 (C-O), 150.2 (N=CH), 132.8 (Ar**C**-C=O), 131.7 (CH), 131.3 (CH), 127.9 (2xCH), 127.3 (2xCH), 110.0 (Ar**C**CH=N), 107.3 (CH), 102.8 (CH) ppm.

(6) **(E)-N'-(2,3,4-trihydroxybenzylidene)benzohydrazide** (**6**) (Smith et al., 2012):- **Yield (%):** 55%; **m.p. °C:** 226–228; **IR:** 3410-3175 (N-H & OH), 3058 (=C-H), 1640 (C=O), 1616 (C=N), 1498 (C=C Ar ring), 1232 (C-O); ^**1**^**H-NMR** (1:1 DMSO-d_6_/CDCl_3_, 400 MHz): δ 11.68 (s, 2H, NH & OH), 8.77 (s,1H, OH), 8.36 (s,1H, C**H** = N), 7.86 (d, *J* = 8.0 Hz, 2H), 7.53–7.35 (m, 3H), 6.60 (d, *J* = 8.0 Hz, 1H), 6.37 (d, *J* = 8.0 Hz, 1H); ^**13**^**C-NMR** (1:1 DMSO-d_6_/CDCl_3_, 100 MHz): δ 162.8 (C=O), 150.3 (N=CH), 148.1 (C-O), 147.2 (C-O), 132.8 (Ar**C**-C=O), 132.3 (C-O), 131.3 (CH), 127.9 (2xCH), 127.3 (2xCH), 121.4 (CH), 110.5 (Ar**C**CH=N), 107.3 (CH) ppm.

**(E)-N'-(4-hydroxy-3-methoxybenzylidene)benzohydrazide** (**7**) (Wang et al., 2014):- **Yield (%):** 74%; **m.p. °C:** 195–197; **IR:** 3490-3255 (N-H & OH), 3060 (=C-H), 1647 (C=O), 1602 (C=N), 1499 (C=C Ar ring), 1280 (C-O), 1200 (C-O); ^**1**^**H-NMR** (1:1 DMSO-d_6_/CDCl_3_, 400 MHz): δ 11.47 (s,1H, NH), 8.71 (s,1H, OH), 8.31 (s,1H, C**H** = N), 7.88 (d, *J* = 8.0 Hz, 2H), 7.72–7.27 (m, 4H), 6.95 (d, *J* = 8.0 Hz, 1H), 6.79 (d, *J* = 8.0 Hz, 1H), 3.78 (s, 3H, OCH_3_); ^**13**^**C-NMR** (1:1 DMSO-d_6_/CDCl_3_, 100 MHz): δ 164.0 (C=O), 149.0 (N=CH), 147.6 (C-O), 133.5 (Ar**C**-C=O), 131.1 (CH), 127.9 (2xCH), 127.4 (2xCH), 125.6 (Ar**C**CH=N), 122.7 (CH), 114.6 (CH), 108.3 (CH), 55.5 (OCH_3_) ppm.

**(E)-N'-(3-methoxybenzylidene)benzohydrazide** (**8**) (Wu et al., 1995):- **Yield (%):** 86%; **m.p. °C:** 194–196; **IR:** 3222 (N-H), 3056 (=C-H), 1647 (C=O), 1600 (C=N), 1545-1451 (C=C Ar ring), 1248 (C-O); ^**1**^**H-NMR** (1:1 DMSO-d_6_/CDCl_3_, 400 MHz): δ 11.11 (s,1H, NH), 8.37 (s, 1H, C**H** = N), 7.57–7.43 (m, 3H), 7.14–6.78 (m, 4H), 6.59–6.37 (m, 2H), 3.34 (s, 3H, OCH_3_); ^**13**^**C-NMR** (1:1 DMSO-d_6_/CDCl_3_, 100 MHz): δ 163.8 (C=O), 157.5 (C-O), 144.0 (N=CH), 133.2 (Ar**C**-C=O), 131.1 (CH), 130.9 (CH), 127.8 (2xCH), 127.4 (2xCH), 126.2 (CH), 122.2 (Ar**C**CH=N), 120.2 (CH), 110.5 (CH), 55.0 (OCH_3_) ppm.

**(E)-2-hydroxy-N'-(2-hydroxybenzylidene)benzohydrazide** (**9**) (Kotali and Lafazanis, 2003):- **Yield (%):** 82%; **m.p. °C:** 284–286; **IR:** 3230 (N-H & OH), 3058 (=C-H), 1634 (C=O), 1608 (C=N), 1554-1490 (C=C Ar ring); ^**1**^**H-NMR** (1:1 DMSO-d_6_/CDCl_3_, 400 MHz): δ 11.89 (s, 1H, NH), 8.51 (s, 1H, C**H** = N), 7.85 (d, *J* = 8.0 Hz, 2H), 7.44–7.17 (m, 3H), 7.00–6.78 (m, 4H); ^**13**^**C-NMR** (1:1 DMSO-d_6_/CDCl_3_, 100 MHz): δ 165.0 (C=O), 160.0 (C-O), 157.8 (C-O), 149.9 (N=CH), 133.8 (CH), 131.0 (CH), 130.0 (CH), 127.6 (CH), 118.8 (CH), 118.4 (CH), 117.7 (Ar**C**CH=N), 117.3 (CH), 116.3 (CH), 113.9 (Ar**C**-C=O) ppm.

**(E)-2-hydroxy-N'-(3-hydroxybenzylidene)benzohydrazide** (**10**) (Rahim et al., 2016):- **Yield (%):** 50%; **m.p. °C:** 253–255; IR: 3270 (N-H), 3070 (C-H), 1647 (C=O), 1585 (C=N),1545-1451 (C=C Ar ring), 1303 (N-N); **IR:** 3230 (N-H & OH), 3058 (=C-H), 1634 (C=O), 1608 (C=N), 1554-1490 (C=C Ar ring); ^**1**^**H-NMR** (1:1 DMSO-d_6_/CDCl_3_, 400 MHz): δ 12.03 (s, 1H, OH, *ortho*), 11.61 (s, 1H, NH), 9.11 (s, 1H, OH), 8.31 (s, 1H, C**H** = N), 7.87 (d, *J* = 8.0 Hz, 1H), 7.39–7.29 (m, 1H), 7.25–7.18 (m, 1H), 7.17–7.06 (m, 2H), 6.95–6.76 (m, 3H); ^**13**^**C-NMR** (1:1 DMSO-d_6_/CDCl_3_, 100 MHz): δ 165.6 (C=O), 160.3 (C-O), 157.2 (C-O), 149.1 (N=CH), 134.7 (Ar**C**CH=N), 133.6 (CH), 129.1 (CH), 127.4 (CH), 118.6 (CH), 118.2 (CH), 117.4 (CH), 117.3 (CH), 114.0 (Ar**C**-C=O), 113.3 (CH) ppm.

**2-Hydroxy(4-hydroxy-benzylidene)benzohydrazide** (**11**) (Jablonski et al., 2012):- **Yield (%):** 51%; **m.p. °C:** 239–241; IR: 3262 (N-H), 1640 (C=O), 1595 (C=N), 1506 (C=C Ar ring), 1220 (C-O); **IR:** 3262 (N-H & OH), 1640 (C=O), 1595 (C=N), 1506 (C=C Ar ring), 1220 (C-O); ^**1**^**H-NMR** (1:1 DMSO-d_6_/CDCl_3_, 400 MHz): δ 12.11 (s, 1H, OH_*ortho*_), 11.51 (s, 1H, NH), 9.44 (s, 1H, OH_*para*_), 8.29 (s, 1H, C**H** = N), 7.87 (d, *J* = 8.0 Hz, 1H), 7.54 (d, *J* = 8.0 Hz, 2H), 7.34 (t, *J* = 8.0 Hz, 1H), 6.89 (d, *J* = 8.0 Hz, 1H), 6.83 (d, *J* = 8.0 Hz, 1H), 6.78 (d, *J* = 8.0 Hz, 2H); ^**13**^**C-NMR** (1:1 DMSO-d_6_/CDCl_3_, 100 MHz): δ 165.5 (C=O), 160.3 (C-O), 155.5 (C-O), 149.5 (N=CH), 133.5 (CH), 128.9 (2xCH), 127.3 (CH), 124.6 (Ar**C**CH=N), 118.2 (CH), 117.3 (CH), 115.3 (2xCH), 114.0 (Ar**C**-C=O) ppm.

**(E)-N'-(3,4-dihydroxybenzylidene)-2-hydroxybenzohydrazide** (**12**) (Alam et al., 2017):**- Yield (%):** 85%; **IR:** 3273 (N-H & OH), 1639 (C=O), 1590 (C=N), 1487 (C=C Ar ring), 1280 (C-O), 1232 (N-N); ^**1**^**H-NMR** (1:1 DMSO-d_6_/CDCl_3_, 400 MHz): δ 12.13 (s, 1H, OH_*ortho*_), 11.45 (s, 1H, NH), 8.77 (s, 1H, OH_*ortho*_*_-para_*), 8.21 (s, 1H, C**H** = N), 7.84 (d, *J* = 8.0 Hz, 1H), 7.39–7.25 (m, 2H), 6.96 (d, *J* = 8.0 Hz, 1H), 6.89 (d, *J* = 8.0 Hz, 1H), 6.81 (t, *J* = 8.0 Hz, 1H), 6.75 (d, *J* = 8.0 Hz, 2H); ^**13**^**C-NMR** (1:1 DMSO-d_6_/CDCl_3_, 100 MHz): δ 165.4 (C=O), 160.3 (C-O), 149.5 (N=CH), 147.6 (C-O), 145.0 (C-O), 133.4 (CH), 127.2 (CH), 125.3 (Ar**C**CH=N), 120.5 (CH), 118.1 (CH), 117.3 (CH), 115.0 (CH), 114.0 (Ar**C**-C=O), 113.3 (CH) ppm.

**(E)-N'-(2,4-dihydroxybenzylidene)-2-hydroxybenzohydrazide** (**13**) (Alam et al., 2017):- **Yield (%):** 55%; **m.p. °C:** 260–262; **IR:** 3426-3247 (N-H & OH), 3066 (C-H), 1632 (C=O), 1600 (C=N), 1499 (C=C Ar ring), 1256 (C-O); ^**1**^**H-NMR** (1:1 DMSO-d_6_/CDCl_3_, 400 MHz): δ 11.98 (s, 1H, OH_*ortho*_), 11.69 (s, 2H, NH & OH), 9.51 (s, 1H, OH), 8.38 (s, 1H, C**H** = N), 7.82 (d, *J* = 8.0 Hz, 1H), 7.38–7.26 (m, 1H), 7.07–6.98 (m, 1H), 6.93–6.76 (m, 2H), 6.41 (d, *J* = 4.0 Hz, 2H); ^**13**^**C-NMR** (1:1 DMSO-d_6_/CDCl_3_, 100 MHz): δ 164.9 (C=O), 160.7 (C-O), 160.3 (C-O), 160.0 (C-O), 150.8 (N=CH), 133.6 (CH), 131.7 (CH), 127.2 (CH), 118.2 (CH), 117.3 (CH), 113.7 (Ar**C**-C=O), 109.8 (Ar**C**CH=N), 107.4 (CH), 102.8 (CH) ppm.

**(E)-2-hydroxy-N'-(2,3,4-trihydroxybenzylidene)benzohydrazide** (**14**) (Alam et al., 2017):- **Yield (%):** 55%; **m.p. °C:** 288-282; IR: 3300 (N-H), 1632 (C=O), 1593 (C=N),1506 (C=C Ar ring), 1303 (C-O), 1249 (C-N); **IR:** 3426-3247 (N-H & OH), 3066 (C=C), 1632 (C=O), 1600 (C=N), 1499 (C=C Ar ring), 1256 (C-O); ^**1**^**H-NMR** (1:1 DMSO-d_6_/CDCl_3_, 400 MHz): δ 12.03 (s, 1H, OH_*ortho*_), 11.72 (s, H, NH), 8.37 (s, 1H, C**H** = N), 7.82 (d, *J* = 8.0 Hz, 1H), 7.40–7.28 (m, 1H), 6.98–6.78 (m, 2H), 6.61 (d, *J* = 4.0 Hz, 1H), 6.46–6.34 (m, 1H); ^**13**^**C-NMR** (1:1 DMSO-d_6_/CDCl_3_, 100 MHz): δ 165.0 (C=O), 160.3 (C-O), 151.1 (N=CH), 148.2 (C-O), 147.2 (C-O), 133.6 (CH), 132.2 (C-O), 127.2 (CH), 121. 6(CH), 118.2 (CH), 117.3 (CH), 113.6 (Ar**C**-C=O), 110.4 (Ar**C**CH=N), 107.3 (CH) ppm.

**(E)-2-hydroxy-N'-(4-hydroxy-3-methoxybenzylidene)benzohydrazide** (**15**) (Wang et al., 2014):- **Yield (%):** 65%; **m.p. °C:** 118–120; **IR:** 2410 (OH), 3254 (N-H), 2925 (C-H), 1647 (C=O), 1600 (C=N), 1506 (C=C Ar ring), 1303 (C-O), 1232 (C-N); ^**1**^**H-NMR** (1:1 DMSO-d_6_/CDCl_3_, 400 MHz): δ 12.08 (s, 1H, OH_*ortho*_), 11.63 (s, 1H, NH), 9.09 (s, 1H, OH_*para*_), 8.31 (s, 1H, C**H** = N), 7.91 (d, *J* = 8.0 Hz, 1H), 7.40–7.30 (m, 2H), 7.02 (d, *J* = 8.0 Hz, 1H), 6.90 (d, *J* = 8.0 Hz, 1H), 6.83 (t, *J* = 8.0 Hz, 2H), 3.84 (s, 3H, OCH_3_); ^**13**^**C-NMR** (1:1 DMSO-d_6_/CDCl_3_, 100 MHz): δ 165.4 (C=O), 160.1 (C-O), 149.8 (N=CH), 149.1 (C-O), 147.7 (C-O), 133.5 (CH), 127.5 (CH), 125.2 (Ar**C**CH=N), 122.7 (CH), 118.3 (CH), 117.3 (CH), 114.9 (CH), 114.2 (Ar**C**-C=O), 108.5 (CH), 55.4 (OCH_3_) ppm.

**(E)-2,4-dihydroxy-N'-(3-hydroxybenzylidene)benzohydrazide** (**16**) (Lai et al., 2017):- **Yield (%):** 40%; **m.p. °C:** 252–254; **IR:** 3520 (OH), 3246 (N-H), 1640 (C=O), 1616 (C=N), 1577-1545 (C=C Ar ring), 1274 (C-O); ^**1**^**H-NMR** (1:1 DMSO-d_6_/CDCl_3_, 400 MHz): δ 12.35 (s, 1H, OH_*ortho*_), 11.39 (s, 1H, NH), 9.74 (s, 1H, OH_meta_), 9.13 (s, 1H, OH_para_), 8.26 (s, 1H, C**H** = N), 7.70 (d, *J* = 8.0 Hz, 1H), 7.18 (s, 1H), 7.16–7.04 (m, 2H), 6.78 (d, *J* = 8.0 Hz, 1H), 6.38–6.21 (m, 2H); ^**13**^**C-NMR** (1:1 DMSO-d_6_/CDCl_3_, 100 MHz): δ 166.0 (C=O), 162.9 (C-O), 162.5 (C-O), 157.3 (C-O), 148.1 (N=CH), 135.0 (Ar**C**CH=N), 129.1 (CH), 128.7 (CH), 118.5 (CH), 117.2 (CH), 113.2 (CH), 107.0 (CH), 105.4 (Ar**C**-C=O), 102.9 (CH) ppm.

**(E)-2,4-dihydroxy-N'-(4-hydroxybenzylidene)benzohydrazide** (**17**) (Lai et al., 2017):- **Yield (%):** 52%; **m.p. °C:** 288-282; **IR**: 3325 (OH), 3270 (N-H), 1632 (C=O), 1608 (C=N), 1506-1436 (C=C Ar ring), 1248 (C-O); ^**1**^**H-NMR** (1:1 DMSO-d_6_/CDCl_3_, 400 MHz): δ 12.42 (s, 1H, OH_*ortho*_), 11.39 (s, 1H, NH), 9.86 (s, 1H, OH_para_), 9.61 (s, 1H, OH_para_), 8.28 (s, 1H, C**H** = N), 7.73 (d, *J* = 8.0 Hz, 1H), 7.51 (d, *J* = 8.0 Hz, 2H), 6.77 (d, *J* = 8.0 Hz, 2H), 6.29 (d, *J* = 8.0 Hz, 1H), 6.27 (s, 1H); ^**13**^**C-NMR** (1:1 DMSO-d_6_/CDCl_3_, 100 MHz): δ 165.7 (C=O), 162.7 (C-O), 162.5 (C-O), 159.4 (C-O), 148.4 (N=CH), 128.8 (CH), 128.7 (2xCH), 124.9 (Ar**C**CH=N), 115.4 (2xCH), 107.0 (CH), 105.4 (Ar**C**-C=O), 102.8 (CH) ppm.

**(E)-N'-(3,4-dihydroxybenzylidene)-2,4-dihydroxybenzohydrazide** (**18**):- **Yield (%):** 45%; **m.p. °C:** 237–239; **IR**: 3300 (OH & N-H), 1640 (C=O),1600 (C=N), 1514 (C=C Ar ring), 1280 (C-O); ^**1**^**H-NMR** (1:1 DMSO-d_6_/CDCl_3_, 400 MHz): δ 12.45 (s, 1H, OH_*ortho*_), 11.22 (s, 1H, NH), 9.68 (s, 1H, OH_para_), 8.18 (s, 1H, C**H** = N), 7.63 (s, 1H), 7.28 (s, 1H), 6.95 (d, *J* = 4.0 Hz, 1H), 6.82–6.72 (m, 2H), 6.37–6.23 (m, 3H); ^**13**^**C-NMR** (1:1 DMSO-d_6_/CDCl_3_, 100 MHz): δ 165.8 (C=O), 162.3 (C-O), 160.3 (C-O), 148.6 (N=CH), 147.4 (C-O), 145.0 (C-O), 128.5 (CH), 125.6 (Ar**C**CH=N), 121.4 (CH), 120.3 (CH), 115.0 (CH), 106.9 (CH), 105.5 (Ar**C**-C=O), 102.9 (CH) ppm.

**(E)-N'-(2,4-dihydroxybenzylidene)-2,4-dihydroxybenzohydrazide** (**19**):- **Yield (%):** 47%; **m.p. °C:** 257–259; **IR:** 3365 (OH), 3255 (N-H), 1625 (C=O), 1570 (C=N), 1506 (C=C Ar ring), 1256 C-O); ^**1**^**H-NMR** (1:1 DMSO-d_6_/CDCl_3_, 400 MHz): δ 11.57 (s, 2H, NH & OH), 8.5–8.0 (br s, 3H, OH), 8.47 (s, 1H, C**H** = N), 7.40–7.38 (m, 2H), 7.06–7.04 (m, 2H), 6.47–6.46 (m, 1H), 6.45–6.44 (m, 1H); ^**13**^**C-NMR** (1:1 DMSO-d_6_/CDCl_3_, 100 MHz): δ 168.4 (C=O), 162.4 (C-O), 149.3 (C-O), 148.0 (N=CH), 144.5 (C-O), 137.1 (C-O), 134.0 (CH), 131.8 (CH), 123.3 (CH), 120.2 (CH), 111.3 (CH), 110.2 (Ar**C**CH=N), 108.8 (CH), 107.8 (Ar**C**-C=O) ppm.

**(E)-3,4,5-trihydroxy-N'-(3-hydroxybenzylidene)benzohydrazide** (**20**) (Taha et al., 2019):- **Yield (%):** 54%; **m.p. °C:** 145–147; IR: 3465 (OH), 3340 (N-H), 1695 (C=O), 1616 (C=N),1460 (C=C Ar ring), 1318 (C-O), 1248 (N-N); ^**1**^**H-NMR** (1:1 DMSO-d_6_/CDCl_3_, 400 MHz): δ 9.45–8.26 (m, 5H), 7.35–6.80 (m,7H); ^**13**^**C-NMR** (1:1 DMSO-d_6_/CDCl_3_, 100 MHz): δ 166.7 (C=O), 161.4 (N=CH), 157.4 (C-O), 145.0 (C-O), 137.7 (C-O), 134.9 (C-O), 129.5 (CH), 120.1 (Ar**C**CH=N), 119.8 (CH), 118.5 (CH), 114.4 (Ar**C**-C=O), 109.0 (2xCH) ppm.

**(2,4-Dihydroxybenzylidene)-3,4,5-trihydroxybenzohydrazide** (**21**) (Carcelli et al., 2016):- **Yield (%):** 49%; **m.p. °C:** 288-282; **IR:** 3457 (OH), 3333 (N-H), 1702 (C=O), 1632 (C=N), 1522-1460 (C=C Ar ring), 1311 (C-O), 1209 (N-N); δ 165.7 (C=O), 162.7 (C-O), 162.5 (C-O), 159.4 (C-O), 148.4 (N=CH), 128.8 (CH), 128.7 (2xCH), 124.9 (Ar**C**CH = N), 115.4 (2xCH), 107.0 (CH), 105.4 (Ar**C**-C=O), 102.8 (CH) ppm; ^**13**^**C-NMR** (1:1 DMSO-d_6_/CDCl_3_, 100 MHz): δ 165.0 (C=O), 158.4 (C-O), 151.0 (N=CH), 148.8 (C-O), 147.9 (C-O), 133.6 (CH), 133.2 (C-O), 127.2 (CH), 125.4 (Ar**C**CH=N), 121. 6(CH), 118.2 (CH), 117.3 (CH), 113.6 (Ar**C**-C=O), 107.0 (CH) ppm.

#### 2.2.3 Biological activity methods

##### 2.2.3.1 Antioxidants activity

a) **2,2-Diphenyl-1-picrylhydrazyl (DDPH) method**

The antioxidant activity assay was conducted following the method of Sharma and Bhat (2009) with slight modifications. Thus, two milliliters of 0.3 mM 2,2-diphenyl-1-picrylhydrazyl solution was mixed with 0.1 mL of hydrazone solution in DMSO (1mg/mL). The reaction mixture was then shaken for a minute and left for further thirty minutes in the dark. The absorbance was measured at a wavelength of 517 nm against a blank. In this case, the well-known Trolox was employed as a positive control. All measurements in this work were carried out in triplicate. In order to calculate the percent (%) of the scavenging activity (%RSA) of the hydrazone sample, the following equation was used:

(%RSA) = [(A_c_ – A_s_) /A_c_] x 100,

where A_c_ represents the absorbance of 2,2-diphenyl-1-picrylhydrazyl in the absence of the test sample and A_s_ stands for the absorbance of 2,2-diphenyl-1-picrylhydrazyl in the presence of the test sample or alternatively, the positive control.

b) **2,2′-Azino-bis(3-Ethylbenzothiazoline-6-Sulfonic Acid (ABTS) Enzymatic Assay**

This enzymatic assay was conducted according to the method described by Arnao et al. (2001) with some modifications as follows: A solution is made by mixing equivalent volumes of 2,2′-azino-bis(3-ethylbenzothiazoline-6-sulfonic acid **(**7.4 mM) solution and potassium persulfate solution (2.6 mM). The mixture was then reacted for fourteen to sixteen hours in the dark at room temperature to produce ABTS^**.+**^. The obtained solution was subsequently diluted by mixing ABTS^**.+**^ solution (1 mL) with pH = 7.4 phosphate buffer solution (60 mL) to reach an absorbance of 1.10 ± 0.02 units at a wavelength of 734 nm using a UV/VIS spectrophotometer. In order to prepare the final ABTS^**.+**^ solution for each assay, the following procedure was carried out: Add 100 **μ**L of hydrazone solution (prepared as 1 mg/mL in DMSO) to 3.0 mL of ABTS^**.+**^ final solution. Incubate for two hours in the dark and measure the absorbance at a wavelength of 734 nm. Trolox (1 mg/mL in DMSO) was used as a positive control. The percent inhibitory rate was worked out according to the following formula:

Percent inhibitory rate (%) = [(A_c_ – A_s_)/ A_c_] x 100

Here, A_c_ is the absorbance of control (without samples) and A_s_ is the absorbance in the presence of the samples.

c) **Inhibition of lipid peroxidation by ferric thiocyanate protocol**

This protocol was employed, with some modifications, to investigate the inhibition of lipid peroxidation as reported by Kikuzaki and Nakatani (1993). Therefore, into a vial with a screw cap (20 mL capacity), transfer hydrazone sample (4 mL made from 1 mg/mL in DMSO), linoleic acid solution (4.1 mL, 2.51%, in absolute ethanol), phosphate buffer (8 mL, pH = 7), and distilled H_2_O (3.9 mL). This mixture was placed in an oven maintained at 40 °C in the dark and was designated as a sample solution. Then:

Transfer 100 **μ**L of a sample solution into a 2 mL screw cap vial, then add 9.7 mL of ethanol (75%), 100 **μ**L **μ**L of NH_4_SCN (30%) and **μ**L of FeCl_2_ (20 mM in 3.5% HCl). After three minutes has passed, the absorbance of the solution (red color) was measured at a wavelength of 500 nm. These measurements were repeated every twenty four hours until one day after the absorbance of the control solution (without sample) has reached its maximum. Ascorbic acid (vitamin C) (1 mg/mL) was used as the positive control in this method. The percent Inhibition (%) of lipid peroxidation was found according to the following equation:

Percent inhibition (%) = [(A_c_ – A_s_) / A_c_] x 100

Where, A_s_ represents the absorbance of the sample or positive control on the day when the absorbance of the control was maximum, and A_c_ represents the absorbance of the control without any sample on the day when it reached its maximum absorbance.

##### 2.2.3.2 Enzymes inhibitions

a) **The inhibition of tyrosinase**

The activity of tyrosinase assay was carried out as previously reported by the group of Chen et al. (2012) with some minor modification. This enzyme was prepared as described by Kuninor et al. (1975). The activity of tyrosinase was measured using l-3,4-dihydroxyphenylalanine as a substrate. Samples were dissolved in dimethyl sulfoxide (1mg/mL), and l-3,4-dihydroxyphenylalanine (2 mM) in 50 mM sodium hydrogen phosphate–sodium dihydrogen phosphate buffer (pH 6.8) were previously incubated at 30 ͦ C. Then, 2.8 mL l-3,4-dihydroxyphenylalanine was mixed with 100 **μ**L sample. After one minute, 100 **μ**L of the aqueous solution of tyrosinase was added to the mixture and the absorbance was immediately measured at a wavelength of 475 nm for seven minutes. All experimental measurements were repeated thrice (performed in triplicate). The percent inhibitory rate was calculated according to the formula:

Percent inhibitory rate = [(A_c_ – A_s_)/ Ac] x 100

Here, A_c_ represents the absorbance of control devoid of samples and A_s_ represents the absorbance in the presence of the samples.

b) **The inhibition of acetylcholinesterase**

Inhibition of acetylcholinesterase (AChE) was measured according to previously described method of Ellman et al. (1961) with minor modifications. In a typical procedure, 2 mL of phosphate-buffered saline (PBS) (Na_2_HPO_3_, 50 mM, pH = 7.7) were taken into a test tube and 100 **μ**L of hydrazone sample dissolved in DMSO (1 mg/mL) was added. Then, 300 **μ**L of enzyme (0.005 U/mL) solution was added to the solution. The solution was incubated at 37 °C for 10 min. Then, 300 **μ**L of acetylthiocholine iodide (0.5 mM, substrate) and 300 **μ**L of Ellman's reagent (5,5-dithio-bis-(2-nitrobenzoic acid, DTNB) (0.5 mM) were added. After 30 min of incubation at 37 °C, absorbance was measured at a wavelength of 412 nm. Each sample was measured in triplicate. The enzyme percent inhibitory rate was calculated according to the following formula:

Percent inhibitory rate = [(A_c_ – A_s_)/ A_c_] x 100

Here, A_c_ represents the absorbance of control devoid of any samples and A_s_ represents the absorbance in the presence of the hydrazone sample.

## 3 Results and discussion

The aromatic hydrazones obtained in the present investigation were tested *in vitro* whether they have direct or indirect (or both) antioxidant activities using different methods, such as, DPPH, ABTS and ferric thiocyanate (FTC).

The direct antioxidant activities of hydrazones as measured by the DPPH method (Table 1) ranged from very low to very high (1.19–89.51). These results have shown that the greater the number of hydroxyl groups (4–5 groups) attached to aromatic rings and also their locations in ortho-positions relative to each other may be responsible for this high biological activity (i.e., compounds **14, 18, 20** and **21**). Some of these compounds showed comparable activity to that obtained from the positive control (Vit. C). On the other hand, the antioxidant activities of the present compounds as measured by the ABTS method, which is also a direct method, were in the range of 68.74–93.51 (Table 1), and most of these compounds have shown to have comparable antioxidant activity to that of the positive control (Trolox). The potential antioxidant activities of these aromatic hydrazones may be as a result of the presence of hydroxyl groups, since the only compound that did not contain any hydroxyl group showed the lowest antioxidant activity (compound **8**). The third indirect method to measure the antioxidant activities of the present hydrazones (Table 1) was employed to investigate the inhibition of lipid peroxidation. The present data indicated that all tested compounds were very efficient as indirect antioxidant agents (85.90–97.60) and even much more active than the positive control (Vit. C). It must be emphasized that aromatic hydrazones containing two neighboring substituents of hydroxyl groups (or *o*-positions, R_2_ = R_3_ = OH) to each other give the highest antioxidant activities according to all used methods (DPPH, ABTS, and FTC) and even more active than those having three hydroxyl groups (R_1_ = R_2_ = R_3_ = OH).

**Table 1:** Antioxidant activities of hydrazones synthesized from benzoic and phenolic acids hydrazides and different aromatic aldehydes.


No.	Name of Compound	DPPH	ABTS	FTC
1	2-hydroxybenzylidene benzohydrazide	1.19 ± 2.20	90.67 ± 0.00	95.30 ± 1.60
2	3-hydroxybenzylidene benzohydrazide	1.19 ± 0.60	90.57 ± 0.09	90.30 ± 1.80
3	4-hydroxybenzylidene benzohydrazide	0.33 ± 0.04	90.67 ± 0.16	95.40 ± 2.10
4	3,4-dihydroxybenzylidene benzohydrazide	70.94 ± 1.60	90.98 ± 0.41	97.50 ± 0.50
5	2,4-dihydroxybenzylidene benzohydrazide	40.68 ± 1.40	91.34 ± 0.09	90.90 ± 0.70
6	2,3,4-trihydroxybenzylidene benzohydrazide	59.87 ± 2.20	89.06 ± 0.09	95.90 ± 2.90
7	4-hydroxy-3-methoxy benzylidene benzohydrazide	64.38 ± 0.60	90.98 ± 0.54	86.30 ± 1.90
8	3-methoxybenzylidene benzohydrazide	3.32 ± 1.50	68.74 ± 2.88	97.10 ± 0.50
9	2-hydroxy(2-hydroxy -benzylidene)benzohydrazide	1.23 ± .11	89.84 ± 1.41	86.40 ± 2.80
10	2-hydroxy (3-hydroxy-benzylidene)benzohydrazide	1.90 ± 0.35	91.14 ± 0.80	91.80 ± 3.20
11	2-hydroxy(4-hydroxy- benzylidene)benzohydrazide	23.40 ± 1.26	90.72 ± 0.09	87.90 ± 1.40
12	(3,4-dihydroxybenzylidene)-2-hydroxybenzohydrazide	48.24 ± 0.160	90.67 ± 0.00	87.80 ± 2.80
13	(2,4-dihydroxybenzylidene)-2-hydroxybenzohydrazide	44.10 ± 0.41	90.20 ± 0 .22	85.90 ± 2.00
14	2-hydroxy(2,3,4-trihydroxy-benzylidene)benzohydrazide	76.05 ± 1.48	93.51 ± 1.32	97.60 ± .00
15	2-hydroxy(3-methoxy -benzylidene)benzohydrazide	36.66 ± 2.36	91.14 ± 0.66	88.70 ± 0.90
16	2,4-dihydroxy(3-hydroxy benzylidene)benzohydrazide	36.66 ± 0.34	90.82 ± .27	85.90 ± 0.20
17	2,4-dihydroxy(4-hydroxy benzylidene)benzohydrazide	36.41 ± 2.07	90.67 ± 0.16	86.20 ± 1.40
18	(3,4-dihydroxybenzylidene)-2,4-dihydroxybenzohydrazide	80.57 ± 0.43	89.74 ± 0.82	90.80 ± 3.20
19	(2,4-dihydroxybenzylidene)-2,4-dihydroxybenzohydrazide	54.93 ± 1.95	90.67 ± 1.00	96.20 ± 2.90
20	3,4,5-trihydroxy(3-hydroxybenzylidene) benzohydrazide	89.51 ± 1.57	90.67 ± 0.00	88.40 ± 0.20
21	(2,4-dihydroxybenzylidene)-3,4,5-trihydroxybenzohydrazide	84.07 ± 0.77	85.30 ± 2.53	93.80 ± 1.50
Vit. C		91.83 ± 1.42	-	51.72 ± 1.11
Trolox		-	94.62 ± 0.64	-

The present data of direct and indirect antioxidant activities as measured by DPPH, ABTS, and FTC methods (Table 1) demonstrate that aromatic hydrazones as promising antioxidant agents, especially those having two hydroxyl (R_2_ = R_3_ = OH) or three hydroxyl groups (R_1_ = R_2_ = R_3_ = OH). Thus, it seems that, the presence of two neighboring hydroxyl groups in this series of aromatic hydrazones (R_2_ = R_3_ = OH) is enough to produce optimum antioxidant activities. The obtained aromatic hydrazones containing greater number of hydroxyl groups are expected to have richer conjugated systems in comparison to those that have a lower number hydroxyl groups. Of course, having multiple hydroxyl substituents attached to both aromatic rings created rich conjugated systems and this factor renders the hydrazones as strong antioxidant agents, because they can easily scavenge free radicals in their solutions by undergoing facile hydrogen radical abstraction (H^**.**^) and becoming themselves free radicals, although more stable than the former free radicals with which they react. The present results are in agreement with other results obtained by other researchers (Alam et al., 2017; Aslam et al., 2016; Al-Mamary et al., 2011) which showed that conjugated systems resulting from aromatic Schiff bases containing multi-hydroxyl groups have much higher antioxidant activities than others.

**Figure 1:**
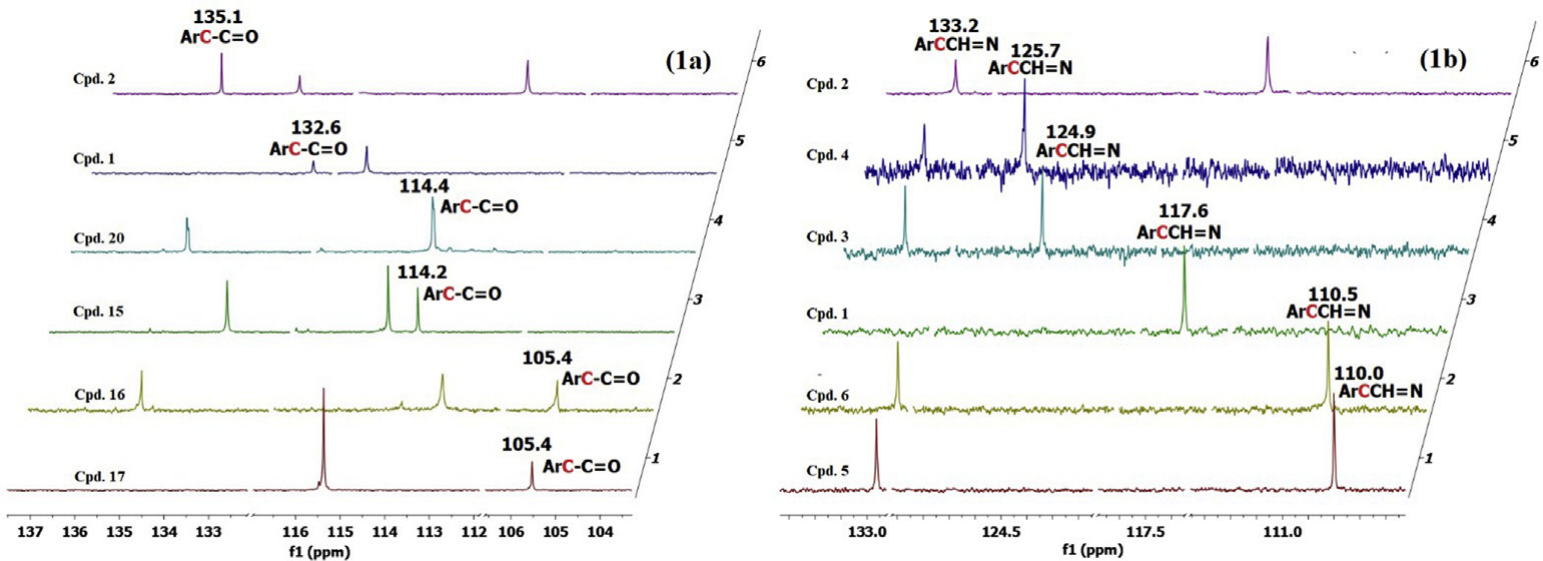
(a) stacked and truncated ^13^C spectra of compounds **1**, **2**, **15**–**17**, and **20** showing the impact of hydroxyl substituents on the carbonyl chemical shifts; (b) stacked and truncated ^13^C spectra of compounds **1**–**6** showing the impact of hydroxyl substituents on the chemical shifts of the quaternary aromatic **C**_**q**_CH = N carbon.

The present compounds with their length of conjugated systems may create and share some common chemical and electronic characteristics similar to those present in natural phenolic compounds (chalcones, stilbenes, and flavonoids). As a result, the explanation of the antioxidant activities of the new synthesized series of aromatic hydrazones is based on that of natural antioxidants such as phenolic compounds. Evidence supporting the strong impact exerted by the hydroxyl groups in delocalizing the aromatic ring electrons within and beyond the aromatic rings is shown in Figure 1 and Table 2. Figure 1a shows a selection of stacked and truncated ^13^C spectra of **1**, **2**, **15**-**7**, and **2** as typical examples demonstrating the strong influence of the hydroxyl groups in delocalizing the electron density within the benzoyl hydrazide ring. Thus, using the ArC-**C**=O group to demonstrate the impact of hydroxyl substituents, the chemical shifts of the quaternary aromatic carbon atom are shown for several compounds in Figure 1a. Clearly, a marked effect has been observed as the chemical shifts ranged from as low as δ105.4 to as high as δ135.1 ppm. On the other hand, the Ar**C**CH = N group was used to gauge the effect of the hydroxyl groups on the delocalization of electrons within the aromatic ring derived from the aldehyde fragment. Similarly, major shift in electron density is noted where the chemical shift values of the aromatic quaternary carbon ranged from δ110.0 to δ135.0 ppm (Figure 1b & Table 2).

**Table 2:** ^**13**^C NMR Chemical shift values for the carbonyl, imine, and non-hydroxylated quaternary carbons of compounds **1-20**.

Cpd. no.	δ (ppm) (C=O)	δ (ppm) (CCH = N)	δ (ppm) ArC-C=O	δ (ppm) (CH = N)
**1**	163.1	117.6	132.6	149.5
**2**	163.8	133.2	135.1	148.2
**3**	163.6	124.9	133.5	148.4
**4**	163.6	125.7	133.5	148.5
**5**	163.0	110.0	132.8	150.2
**6**	162.8	110.5	132.8	150.3
**7**	164.0	125.6	133.5	149.0
**8**	163.8	122.2	133.2	144.0
**9**	165.0	117.7	113.9	149.9
**10**	165.6	134.7	114.0	149.1
**11**	165.5	124.6	114.0	149.5
**12**	165.4	125.3	114.0	149.5
**13**	164.9	109.8	113.7	150.8
**14**	165.0	110.4	113.6	151.1
**15**	165.4	125.2	114.2	149.8
**16**	166.0	135.0	105.4	148.1
**17**	165.7	124.9	105.4	148.4
**18**	165.8	125.9	105.5	148.6
**19**	168.4	110.2	107.8	148.0
**20**	166.7	120.1	114.4	161.4

In general, the efficiency of the antioxidant activity is not only related to delocalizing the free radical within aromatic rings, but also outside the rings. Many natural antioxidants exhibit extended conjugation as key features. Thus, in order to examine whether the hydroxyl substituents impact groups outside the rings, the chemical shifts of the carbonyl (C=O) and imine (CH = N) moieties were collected and are summarized in Table 2. While the carbonyl chemical shifts for **1**–**20** ranged from δ168.4–162.8 ppm, the imine group was impacted to a greater extent (δ161.4–148.0 ppm).

**Figure 2:**
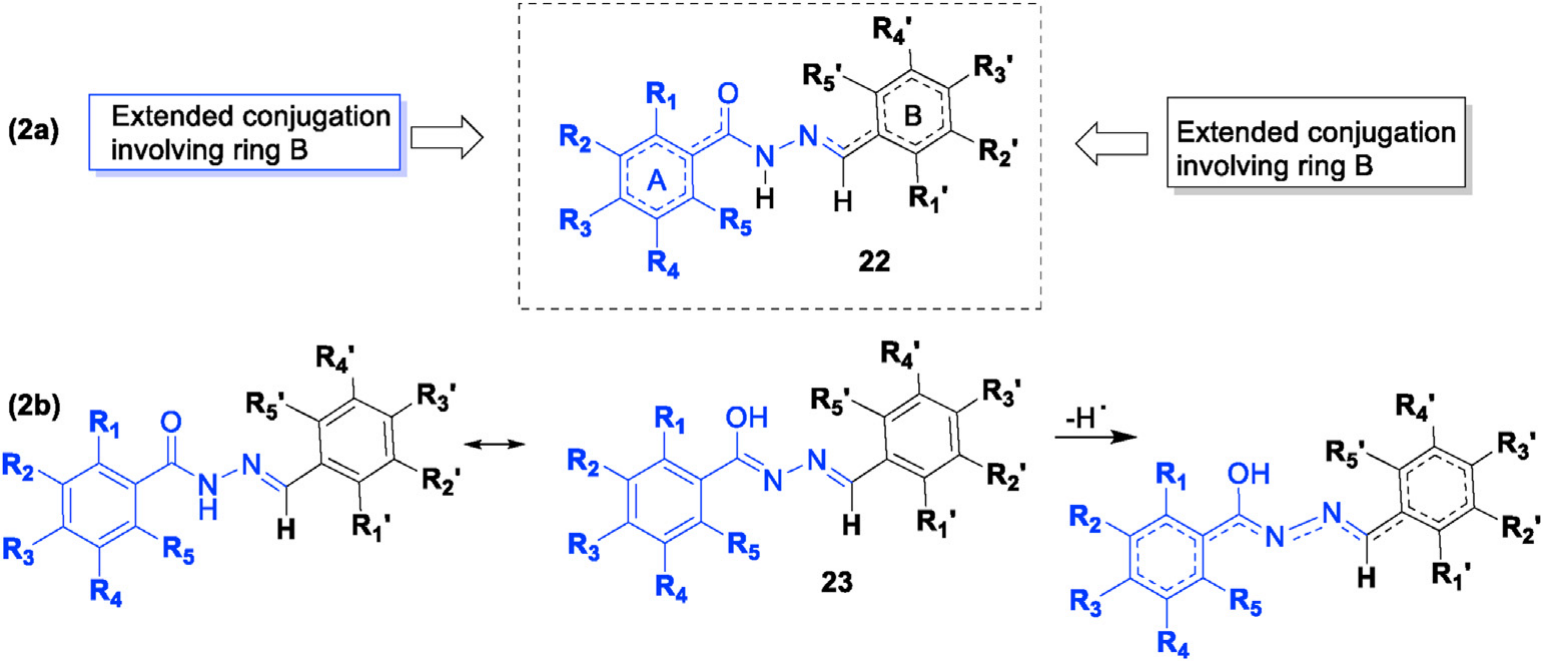
(a) Extended conjugation from both aromatic rings; (b) possible extended conjugation throughout the entire molecule.

These above data support extended conjugation from both aromatic ring as shown in Figure 2 (structure **22**). Although a detailed mechanistic study warrants further investigation, it is conceivable that extended conjugation throughout the entire aromatic hydrazone framework may be feasible considering the possible tautomeric structure **23** shown in Figure 2. Alternatively, radical mobility between the aromatic rings may be possible via intramolecular hydrogen abstraction of the hydrazone NH by a hydroxyl radical formed at R_5_ or from an intermolecular hydrogen abstraction by any of the hydroxyl groups on the other aromatic ring.

It is well known that the generation of reactive oxygen species (ROS) due to metabolic processes leads to various serious diseases such as Alzheimer's disease (AD) and cause skin ageing. There is enough evidence in the literature to relate oxidative stress caused by ROS activity to age-related neurodegenerative ailments and AD. The use of antioxidants has been investigated in an effort to slow down the progression of such diseases (Adewusi et al., 2012). This motivated us to contribute to this area of research. As a result, it was one of our objectives to test the ability of the synthesized aromatic hydrazones to inhibit the tyrosinase and cholinesterase activities hoping to find out new compounds that can be used in cosmetics to prevent or resist melanoma (skin cancer) and to treat AD. The high activities of these two enzymes are used as markers to indicate melanoma and AD.

Generally, enzymes can be inhibited by a substance known as enzyme inhibitor which normally reduces the activity of the enzyme by any one of the known mechanisms. Therefore, each one of the present hydrazones may exert different inhibition effect on either tyrosinase or cholinesterase activity. As the nature and the active sites of enzymes are usually different from each other, so the inhibitors of tyrosinase and cholinesterase are expected to be different.

The results summarizing the inhibition effects of the aromatic hydrazones on the activities of tyrosinase and cholinesterase are shown in Table 3. The inhibition of tyrosinase and cholinesterase by the present series of aromatic hydrazones were in the range of 7.74–66.70 and 0.33–96.99, respectively (Table 3). If we exclude compound **5**, almost all other compounds affected both enzymes differently and these are considered to be normal as expected. The new data have shown that tyrosinase is moderately inhibited (50.54–62.90) by compounds **5**, **6**, and **18** (Table 3) at the level used. On the other hand, the activity of cholinesterase was inhibited moderately to very high (60.48–96.99) by four compounds **(5**, **16**, **17**, and **18**; Table 3). Therefore, compounds **5** and **18** can be considered as promising agents for treatment skin cancer and AD disease.

**Table 3:** The inhibition of tyrosinase and cholinesterase by hydrazones synthesized from benzoic and phenolic acids hydrazides and different aromatic aldehydes.


No.	Name of Compound	Tyrosinase Inhibition (%)	Cholinesterase Inhibition (%)
1	2-hydroxybenzylidene benzohydrazide	47.42 ± 1.37	0.33 ± 1.61
2	3-hydroxybenzylidene benzohydrazide	41.61 ± .46	26.23 ± 1.78
3	4-hydroxybenzylidene benzohydrazide	38.71 ± 2.96	30.91 ± 1.63
4	3,4-dihydroxy benzylidene benzohydrazide	33.87 ± 1.37	8.35 ± 1.88
5	2,4-trihydroxy benzylidene benzohydrazide	62.90 ± .1.37	65.50 ± 3.15
6	2,3,4-trihydroxy benzylidene benzohydrazide	50.54 ± 3.72	35.21 ± 1.24
7	4-hydroxy-3-methoxy benzylidene benzohydrazide	43.66 ± 4.15	12.03 ± 3.54
8	3-methoxybenzylidene benzohydrazide	35.81 ± 3.19	15.54 ± 3.54
9	2-hydroxy(2-hydroxy -benzylidene)benzohydrazide	21.29 ± 1.82	34.67 ± .14
10	2-hydroxy (3-hydroxy-benzylidene)benzohydrazide	11.18 ± 3.19	18.13 ± 2.33
11	2-hydroxy(4-hydroxy- benzylidene)benzohydrazide	32.47 ± 2.91	44.03 ± 2.09
12	(3,4-dihydroxybenzylidene)-2-hydroxybenzohydrazide	36.45 ± 2.91	22.16 ± .18
13	(2,4-dihydroxybenzylidene)-2-hydroxybenzohydrazide	42.58 ± 2.23	21.80 ± 1.59
14	2-hydroxy(2,3,4-trihydroxy -benzylidene)benzohydrazide	40.00 ± 3.23	22.89 ± 2.76
15	2-hydroxy(3-methoxy-benzylidene)benzohydrazide	43.01 ± 2.69	28.07 ± 3.19
16	2,4-dihydroxy(3-hydroxy benzylidene)benzohydrazide	27.31 ± 1.34	96.99 ± 2.14
17	2,4-dihydroxy(4-hydroxy benzylidene)benzohydrazide	21.94 ± 0.65	60.48 ± 2.13
18	(3,4-dihydroxybenzylidene)-2,4dihydroxybenzohydrazide	66.70 ± 2.91	87.89 ± 0.58
19	(2,4-dihydroxybenzylidene)-2,4-dihydroxybenzohydrazide	34.62 ± 1.97	52.63 ± 1.74
20	3,4,5-trihydroxy(3-hydroxybenzylidene) benzohydrazide	28.06 ± 4.11	8.27 ± 1.77
21	(2,4-dihydroxybenzylidene)-3,4,5-trihydroxybenzohydrazide	7.74 ± 2.81	36.59 ± 2.48

## 4 Conclusion

In the present study, a series of 21 aromatic hydrazones were prepared and their chemical structures confirmed by infrared, ^1^H-NMR, and ^13^C-NMR spectroscopy. The compounds contained different number of hydroxyl groups attached to aromatic rings. All compounds were assessed *in vitro* for their antioxidant activity and their ability to inhibit enzymes related to oxidative stress namely tyrosinase and cholinesterase. The new findings have shown that some of the synthesized compounds as antioxidant were comparable and even better than the positive control used in this study (Vit. C and Trolox). In addition, some of the prepared compounds proved as effective inhibitors of tyrosinase and cholinesterase enzymes.

## Declarations

### Author contribution statement

Ziad Moussa: Analyzed and interpreted the data; Wrote the paper.

Mohammed Al-Mamary: Conceived and designed the experiments; Analyzed and interpreted the data; Wrote the paper.

Sultan Al-Juhani, Saleh A Ahmed: Performed the experiments.

### Funding statement

Ziad Moussa was supported by 10.13039/501100006013United Arab Emirates University (Grant no. G00003291/Fund no.31S401/Project #852).

### Competing interest statement

The authors declare no conflict of interest.

### Additional information

No additional information is available for this paper.

## Appendix A Supplementary data

SI 09102020.docx SI 09102020.docx
