# Supplementary material for: Preparation and biological assessment of some aromatic hydrazones derived from hydrazides of phenolic acids and aromatic aldehydes
Source: Heliyon. 2020 Sep 29;6(9):e05019. doi: 10.1016/j.heliyon.2020.e05019 (PMC7527643; doi:10.1016/j.heliyon.2020.e05019)
Supplement: SI 09102020.docx [file mmc1.docx]

# Supporting Information

**Preparation and Biological Assessment of Some Aromatic Hydrazones Derived from Hydrazides of Phenolic Acids and Aromatic Aldehydes**

Ziad Moussa*^1^, Mohammed Al-Mamary*^2^, Sultan Al-Juhani^2^, Saleh A. Ahmed^3,4^

^1^Department of Chemistry, College of Science, United Arab Emirates University, P.O. Box 15551, Al Ain, United Arab Emirates

^2^Chemistry Department, Faculty of Science, Taibah University, PO Box 30002, Code, 14177, Al-Madinah Al-Almunawarrah, Kingdom of Saudi Arabia

^3^Chemistry Department of Chemistry, Faculty of Applied Science, Umm Al-Qura University, 21955 Makkah, Saudi Arabia.

^4^Department of Chemistry, Faculty of Science, Assiut University, 71516 Assiut, Egypt.

Correspondence may be addressed to:

Ziad Moussa: E-mail address: [zmoussa@uaeu.ac.ae](file:///C:\Users\zmoussa\Desktop\sultan%20paper%20latest%20documents\submission%20files%20from%20ma3marry%204152020\zmoussa@uaeu.ac.ae)

^1^H, ^13^C, and IR spectra for 1-20 are provided.

Table of Contents: page

| **^1^H NMR Spectrum of (E)-N'-(2-hydroxybenzylidene)benzohydrazide (1)** | **^S6^** |
| --- | --- |
| **^13^C DEPT 90 NMR Spectrum of (E)-N'-(2-hydroxybenzylidene)benzohydrazide (1)** | **^S7^** |
| **^13^C NMR Spectrum of (E)-N'-(2-hydroxybenzylidene)benzohydrazide (1)** | **^S8^** |
| **IR Spectrum of (E)-N'-(2-hydroxybenzylidene)benzohydrazide (1)** | **^S9^** |
| **^1^H NMR Spectrum of (E)-N'-(3-hydroxybenzylidene)benzohydrazide (2)** | **^S10^** |
| **^13^C DEPT 90 NMR Spectrum of (E)-N'-(3-hydroxybenzylidene)benzohydrazide (2)** | **^S11^** |
| **^13^C NMR Spectrum of (E)-N'-(3-hydroxybenzylidene)benzohydrazide (2)** | **^S12^** |
| **IR Spectrum of (E)-N'-(3-hydroxybenzylidene)benzohydrazide (2)** | **^S13^** |
| **^1^H NMR Spectrum of (E)-N'-(4-hydroxybenzylidene)benzohydrazide (3)** | **^S14^** |
| **^13^C DEPT 90 NMR Spectrum of (E)-N'-(4-hydroxybenzylidene)benzohydrazide (3)** | **^S15^** |
| **^13^C NMR Spectrum of (E)-N'-(4-hydroxybenzylidene)benzohydrazide (3)** | **^S16^** |
| **IR Spectrum of (E)-N'-(4-hydroxybenzylidene)benzohydrazide (3)** | **^S17^** |
| **^1^H NMR Spectrum of (E)-N'-(3,4-dihydroxybenzylidene)benzohydrazide (4)** | **^S18^** |
| **^13^C DEPT 90 NMR Spectrum of (E)-N'-(3,4-dihydroxybenzylidene)benzohydrazide (4)** | **^S19^** |
| **^13^C NMR Spectrum of (E)-N'-(3,4-dihydroxybenzylidene)benzohydrazide (4)** | **^S20^** |
| **IR Spectrum of (E)-N'-(3,4-dihydroxybenzylidene)benzohydrazide (4)** | **^S21^** |
| **^1^H NMR Spectrum of (E)-N'-(2,4-dihydroxybenzylidene)benzohydrazide (5)** | **^S22^** |
| **^13^C DEPT 90 NMR Spectrum of (E)-N'-(2,4-dihydroxybenzylidene)benzohydrazide (5)** | **^S23^** |
| **^13^C NMR Spectrum of (E)-N'-(2,4-dihydroxybenzylidene)benzohydrazide (5)** | **^S24^** |
| **IR Spectrum of (E)-N'-(2,4-dihydroxybenzylidene)benzohydrazide (5)** | **^S25^** |
| **^1^H NMR Spectrum of (E)-N'-(2,3,4-trihydroxybenzylidene)benzohydrazide (6)** | **^S26^** |
| **^13^C DEPT 90 NMR Spectrum of (E)-N'-(2,3,4-trihydroxybenzylidene)benzohydrazide (6)** | **^S27^** |
| **^13^C NMR Spectrum of (E)-N'-(2,3,4-trihydroxybenzylidene)benzohydrazide (6)** | **^S28^** |
| **IR Spectrum of (E)-N'-(2,3,4-trihydroxybenzylidene)benzohydrazide (6)** | **^S29^** |
| **^1^H NMR Spectrum of (E)-N'-(4-hydroxy-3-methoxybenzylidene)benzohydrazide (7)** | **^S30^** |
| **^13^C DEPT 90 NMR Spectrum of (E)-N'-(4-hydroxy-3 methoxybenzylidene)benzohydrazide (7)** | **^S31^** |
| **^13^C NMR Spectrum of (E)-N'-(4-hydroxy-3-methoxybenzylidene)benzohydrazide (7)** | **^S32^** |
| **IR Spectrum of (E)-N'-(4-hydroxy-3-methoxybenzylidene)benzohydrazide (7)** | **^S33^** |
| **^1^H NMR Spectrum of (E)-N'-(3-methoxybenzylidene)benzohydrazide (8)** | **^S34^** |
| **^13^C DEPT 90 NMR Spectrum of (E)-N'-(3-methoxybenzylidene)benzohydrazide (8)** | **^S35^** |
| **^13^C NMR Spectrum of (E)-N'-(3-methoxybenzylidene)benzohydrazide (8)** | **^S36^** |
| **IR Spectrum of (E)-N'-(3-methoxybenzylidene)benzohydrazide (8)** | **^S37^** |
| **^1^H NMR Spectrum of (E)-2-hydroxy-N'-(2-hydroxybenzylidene)benzohydrazide (9)** | **^S38^** |
| **^13^C DEPT 90 NMR Spectrum of (E)-2-hydroxy-N'-(2-hydroxybenzylidene)benzohydrazide (9)** | **^S39^** |
| **^13^C NMR Spectrum of (E)-2-hydroxy-N'-(2-hydroxybenzylidene)benzohydrazide (9)** | **^S40^** |
| **IR Spectrum of (E)-2-hydroxy-N'-(2-hydroxybenzylidene)benzohydrazide (9)** | **^S41^** |
| **^1^H NMR Spectrum of (E)-2-hydroxy-N'-(3-hydroxybenzylidene)benzohydrazide (10)** | **^S42^** |
| **^13^C DEPT 90 NMR Spectrum of (E)-2-hydroxy-N'-(3-hydroxybenzylidene)benzohydrazide (10)** | **^S43^** |
| **^13^C NMR Spectrum of (E)-2-hydroxy-N'-(3-hydroxybenzylidene)benzohydrazide (10)** | **^S44^** |
| **IR Spectrum of (E)-2-hydroxy-N'-(3-hydroxybenzylidene)benzohydrazide (10)** | **^S45^** |
| **^1^H NMR Spectrum of (E)-2-hydroxy-N'-(4-hydroxybenzylidene)benzohydrazide (11)** | **^S46^** |
| **^13^C DEPT 90 NMR Spectrum of (E)-2-hydroxy-N'-(4-hydroxybenzylidene)benzohydrazide (11)** | **^S47^** |
| **^13^C NMR Spectrum of (E)-2-hydroxy-N'-(4-hydroxybenzylidene)benzohydrazide (11)** | **^S48^** |
| **IR Spectrum of (E)-2-hydroxy-N'-(4-hydroxybenzylidene)benzohydrazide (11)** | **^S49^** |
| **^1^H NMR Spectrum of (E)-N'-(3,4-dihydroxybenzylidene)-2-hydroxybenzohydrazide (12)** | **^S50^** |
| **^13^C DEPT 90 NMR Spectrum of (E)-N'-(3,4-dihydroxybenzylidene)-2-hydroxybenzohydrazide (12)** | **^S51^** |
| **^13^C NMR Spectrum of (E)-N'-(3,4-dihydroxybenzylidene)-2-hydroxybenzohydrazide (12)** | **^S52^** |
| **IR Spectrum of (E)-N'-(3,4-dihydroxybenzylidene)-2-hydroxybenzohydrazide (12)** | **^S53^** |
| **^1^H NMR Spectrum of (E)-N'-(2,4-dihydroxybenzylidene)-2-hydroxybenzohydrazide (13)** | **^S54^** |
| **^13^C DEPT 90 NMR Spectrum of (E)-N'-(2,4-dihydroxybenzylidene)-2-hydroxybenzohydrazide (13)** | **^S55^** |
| **^13^C NMR Spectrum of (E)-N'-(2,4-dihydroxybenzylidene)-2-hydroxybenzohydrazide (13)** | **^S56^** |
| **IR Spectrum of (E)-N'-(2,4-dihydroxybenzylidene)-2-hydroxybenzohydrazide (13)** | **^S57^** |
| **^1^H NMR Spectrum of (E)-2-hydroxy-N'-(2,3,4-trihydroxybenzylidene)benzohydrazide (14)** | **^S58^** |
| **^13^C DEPT 90 NMR Spectrum of (E)-2-hydroxy-N'-(2,3,4-trihydroxybenzylidene)benzohydrazide (14)** | **^S59^** |
| **^13^C NMR Spectrum of (E)-2-hydroxy-N'-(2,3,4-trihydroxybenzylidene)benzohydrazide (14)** | **^S60^** |
| **IR Spectrum of (E)-2-hydroxy-N'-(2,3,4-trihydroxybenzylidene)benzohydrazide (14)** | **^S61^** |
| **^1^H NMR Spectrum of (E)-2-hydroxy-N'-(4-hydroxy-3-methoxybenzylidene)benzohydrazide (15)** | **^S62^** |
| **^13^C DEPT 90 NMR Spectrum of (E)-2-hydroxy-N'-(4-hydroxy-3-methoxybenzylidene)benzohydrazide (2m should be corrected to 2k)** | **^S63^** |
| **^13^C NMR Spectrum of (E)-2-hydroxy-N'-(4-hydroxy-3-methoxybenzylidene)benzohydrazide (15)** | **^S64^** |
| **IR Spectrum of (E)-2-hydroxy-N'-(4-hydroxy-3-methoxybenzylidene)benzohydrazide (15)** | **^S65^** |
| **^1^H NMR Spectrum of (E)-2,4-dihydroxy-N'-(3-hydroxybenzylidene)benzohydrazide (16)** | **^S66^** |
| **^13^C DEPT 90 NMR Spectrum of (E)-2,4-dihydroxy-N'-(3-hydroxybenzylidene)benzohydrazide (16)** | **^S67^** |
| **^13^C NMR Spectrum of (E)-2,4-dihydroxy-N'-(3-hydroxybenzylidene)benzohydrazide (16)** | **^S68^** |
| **IR Spectrum of (E)-2,4-dihydroxy-N'-(3-hydroxybenzylidene)benzohydrazide (16)** | **^S69^** |
| **^1^H NMR Spectrum of (E)-2,4-dihydroxy-N'-(4-hydroxybenzylidene)benzohydrazide (17)** | **^S70^** |
| **^13^C DEPT 90 NMR Spectrum of (E)-2,4-dihydroxy-N'-(4-hydroxybenzylidene)benzohydrazide (17)** | **^S71^** |
| **^13^C NMR Spectrum of (E)-2,4-dihydroxy-N'-(4-hydroxybenzylidene)benzohydrazide (17)** | **^S72^** |
| **IR Spectrum of (E)-2,4-dihydroxy-N'-(4-hydroxybenzylidene)benzohydrazide (17)** | **^S73^** |
| **^1^H NMR Spectrum of (E)-N'-(3,4-dihydroxybenzylidene)-2,4-dihydroxybenzohydrazide (18)** | **^S74^** |
| **^13^C DEPT 90 NMR Spectrum of (E)-N'-(3,4-dihydroxybenzylidene)-2,4-dihydroxybenzohydrazide (18)** | **^S75^** |
| **^13^C NMR Spectrum of (E)-N'-(3,4-dihydroxybenzylidene)-2,4-dihydroxybenzohydrazide (18)** | **^S76^** |
| **IR Spectrum of (E)-N'-(3,4-dihydroxybenzylidene)-2,4-dihydroxybenzohydrazide (18)** | **^S77^** |
| **^1^H NMR Spectrum of (E)-N'-(2,4-dihydroxybenzylidene)-2,4-dihydroxybenzohydrazide (19)** | **^S78^** |
| **^13^C NMR Spectrum of (E)-N'-(2,4-dihydroxybenzylidene)-2,4-dihydroxybenzohydrazide (19)** | **^S79^** |
| **IR Spectrum of (E)-N'-(2,4-dihydroxybenzylidene)-2,4-dihydroxybenzohydrazide (19)** | **^S80^** |
| **^1^H NMR Spectrum of (E)-3,4,5-trihydroxy-N'-(3-hydroxybenzylidene)benzohydrazide (20)** | **^S81^** |
| **^13^C DEPT 90 NMR Spectrum of (E)-3,4,5-trihydroxy-N'-(3-hydroxybenzylidene)benzohydrazide (20)** | **^S82^** |
| **^13^C NMR Spectrum of (E)-3,4,5-trihydroxy-N'-(3-hydroxybenzylidene)benzohydrazide (20)** | **^S83^** |
| **IR Spectrum of (E)-3,4,5-trihydroxy-N'-(3-hydroxybenzylidene)benzohydrazide (20)** | **^S84^** |

**^1^H NMR Spectrum of (E)-N'-(2-hydroxybenzylidene)benzohydrazide (1)**

**^13^C DEPT 90 NMR Spectrum of (E)-N'-(2-hydroxybenzylidene)benzohydrazide (1)**

**^13^C NMR Spectrum of (E)-N'-(2-hydroxybenzylidene)benzohydrazide (1)**

**IR Spectrum of (E)-N'-(2-hydroxybenzylidene)benzohydrazide (1)**

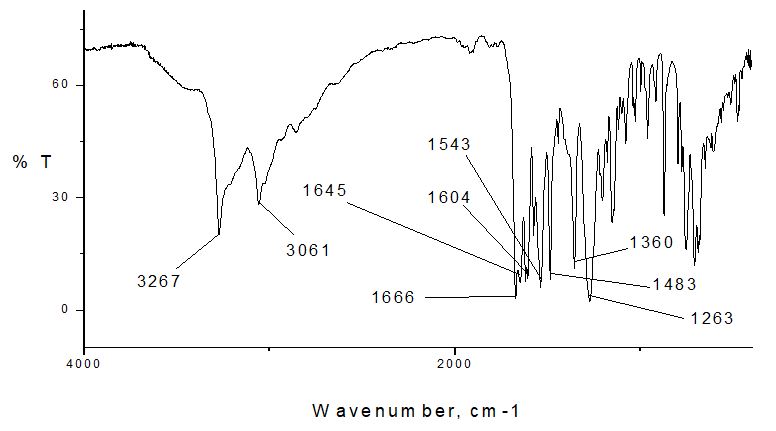


**^1^H NMR Spectrum of (E)-N'-(3-hydroxybenzylidene)benzohydrazide (2)**

**^13^C DEPT 90 NMR Spectrum of (E)-N'-(3-hydroxybenzylidene)benzohydrazide (2)**

**^13^C NMR Spectrum of (E)-N'-(3-hydroxybenzylidene)benzohydrazide (2)**

**IR Spectrum of (E)-N'-(3-hydroxybenzylidene)benzohydrazide (2)**

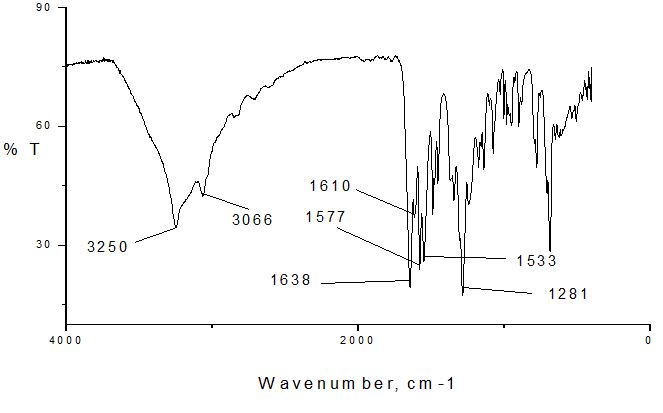


**^1^H NMR Spectrum of (E)-N'-(4-hydroxybenzylidene)benzohydrazide (3)**

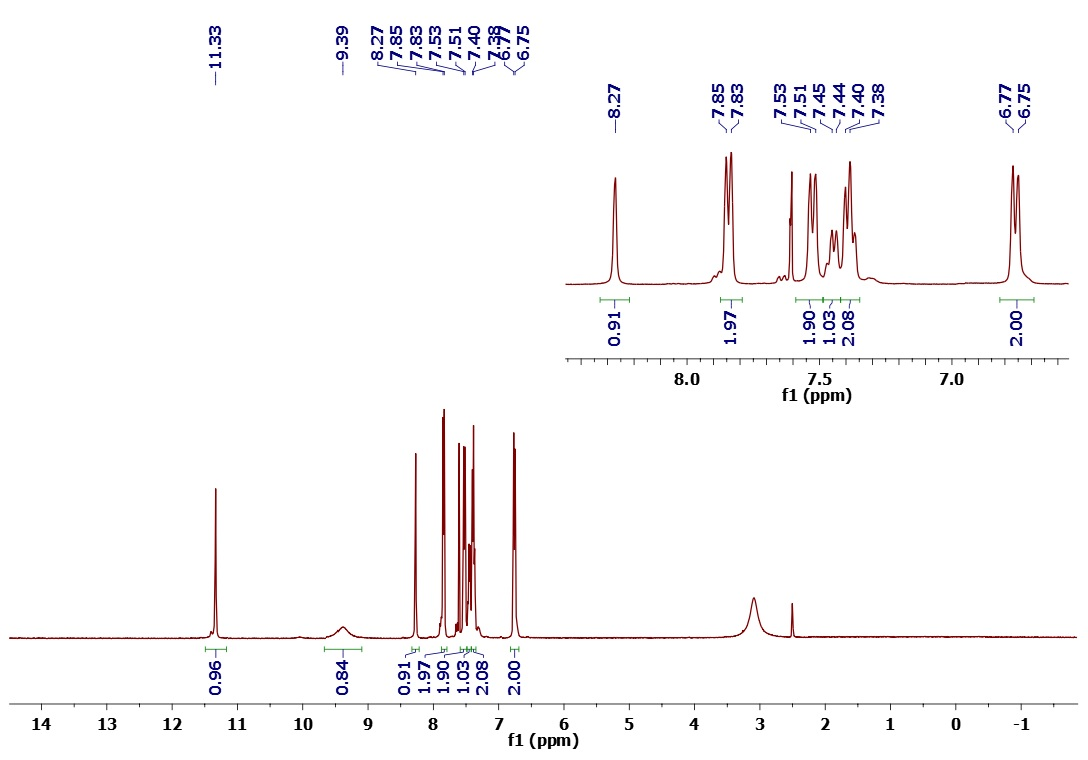


**^13^C DEPT 90 NMR Spectrum of (E)-N'-(4-hydroxybenzylidene)benzohydrazide (3)**

**^13^C NMR Spectrum of (E)-N'-(4-hydroxybenzylidene)benzohydrazide (3)**

**IR Spectrum of (E)-N'-(4-hydroxybenzylidene)benzohydrazide (3)**

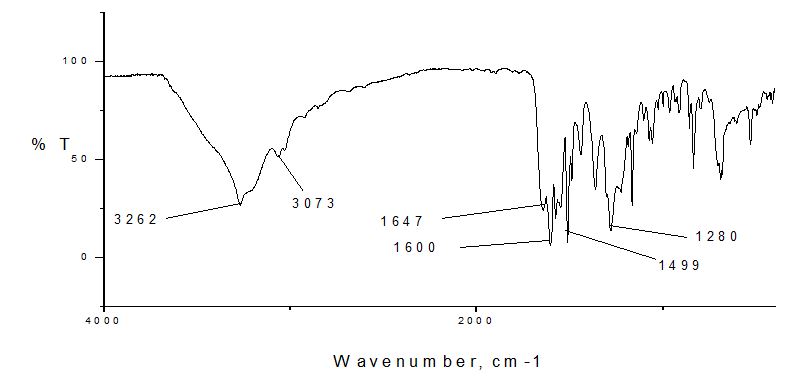


**^1^H NMR Spectrum of (E)-N'-(3,4-dihydroxybenzylidene)benzohydrazide (4)**

**^^**

**^13^C DEPT 90 NMR Spectrum of (E)-N'-(3,4-dihydroxybenzylidene)benzohydrazide (4)**

**^13^C NMR Spectrum of (E)-N'-(3,4-dihydroxybenzylidene)benzohydrazide (4)**

**IR Spectrum of (E)-N'-(3,4-dihydroxybenzylidene)benzohydrazide (4)**

**
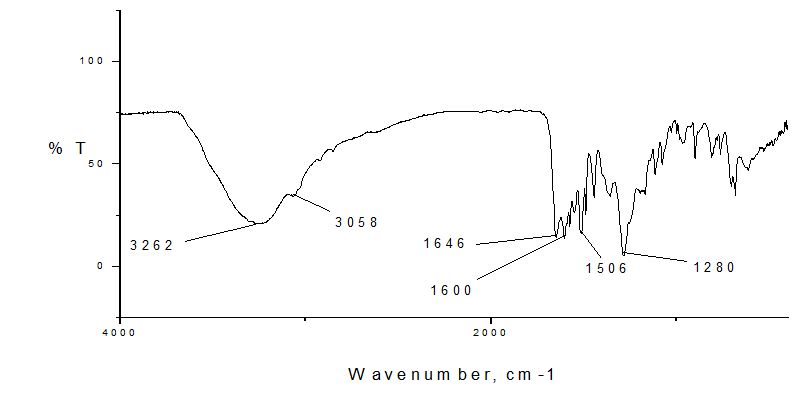
**

**^1^H NMR Spectrum of (E)-N'-(2,4-dihydroxybenzylidene)benzohydrazide (5)**

**^13^C DEPT 90 NMR Spectrum of (E)-N'-(2,4-dihydroxybenzylidene)benzohydrazide (5)**

**^13^C NMR Spectrum of (E)-N'-(2,4-dihydroxybenzylidene)benzohydrazide (5)**

**IR Spectrum of (E)-N'-(2,4-dihydroxybenzylidene)benzohydrazide (5)**

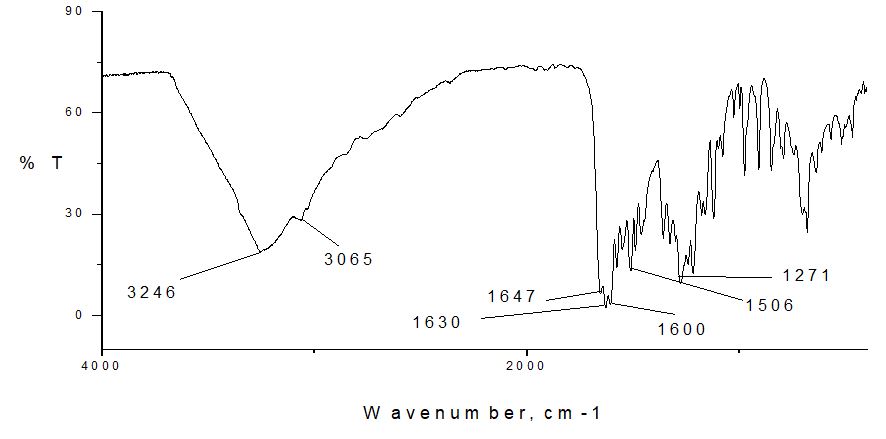


**^1^H NMR Spectrum of (E)-N'-(2,3,4-trihydroxybenzylidene)benzohydrazide (6)**

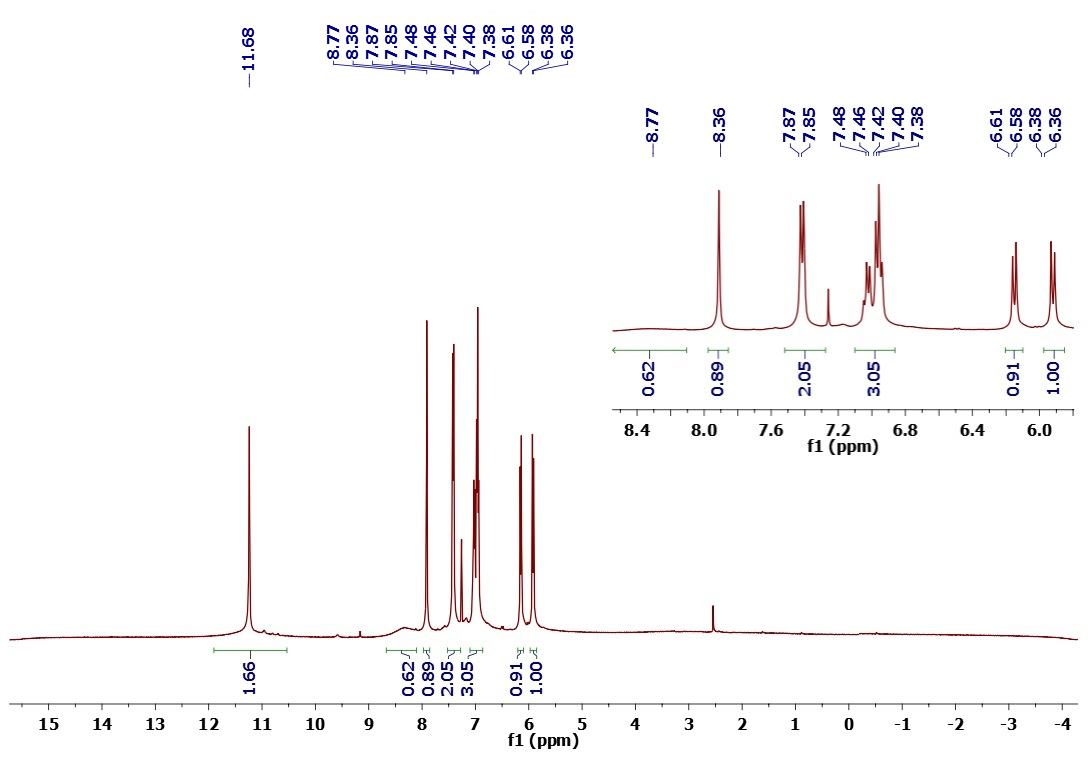


**^13^C DEPT 90 NMR Spectrum of (E)-N'-(2,3,4-trihydroxybenzylidene)benzohydrazide (6)**

**^13^C NMR Spectrum of (E)-N'-(2,3,4-trihydroxybenzylidene)benzohydrazide (6)**

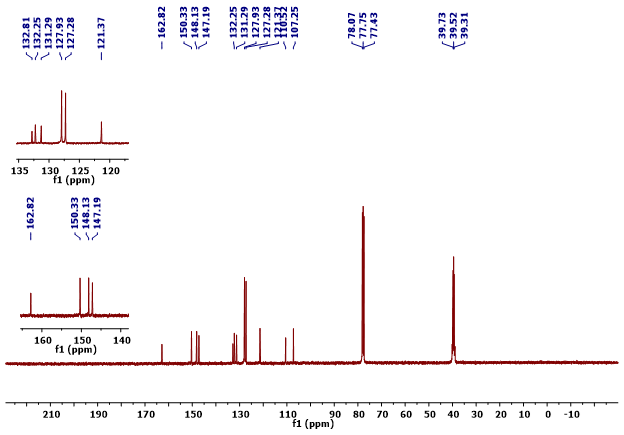


**IR Spectrum of (E)-N'-(2,3,4-trihydroxybenzylidene)benzohydrazide (6)**

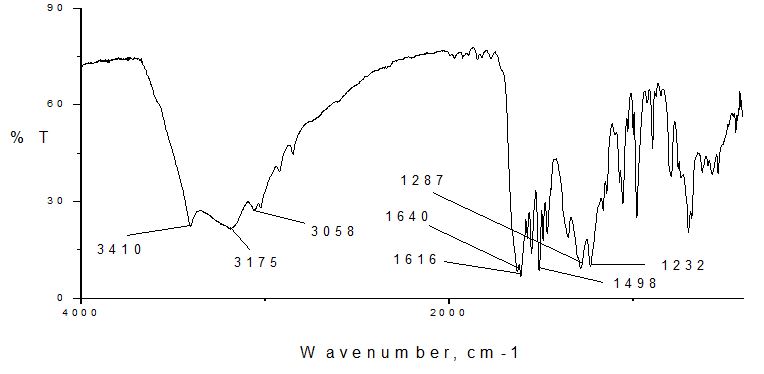


**^1^H NMR Spectrum of (E)-N'-(4-hydroxy-3-methoxybenzylidene)benzohydrazide (7)**

**^^**

**^13^C DEPT 90 NMR Spectrum of (E)-N'-(4-hydroxy-3-methoxybenzylidene)benzohydrazide (7)**

**^^**

**^13^C NMR Spectrum of (E)-N'-(4-hydroxy-3-methoxybenzylidene)benzohydrazide (7)**

**IR Spectrum of (E)-N'-(4-hydroxy-3-methoxybenzylidene)benzohydrazide (7)**

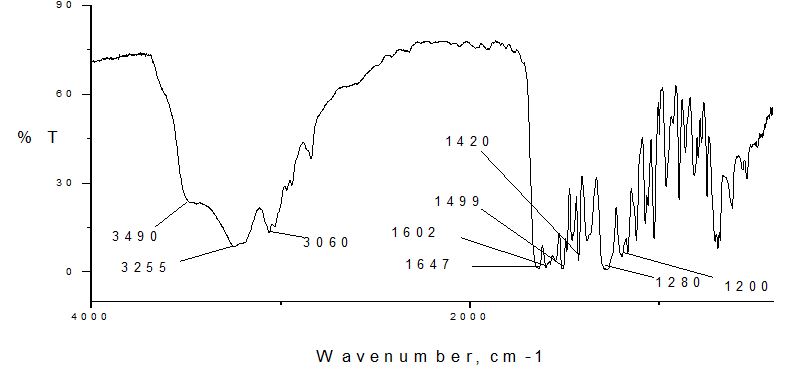


**^1^H NMR Spectrum of (E)-N'-(3-methoxybenzylidene)benzohydrazide (8)**

**^13^C DEPT 90 NMR Spectrum of (E)-N'-(3-methoxybenzylidene)benzohydrazide (8)**

**^13^C NMR Spectrum of (E)-N'-(3-methoxybenzylidene)benzohydrazide (8)**

**IR Spectrum of (E)-N'-(3-methoxybenzylidene)benzohydrazide (8)**

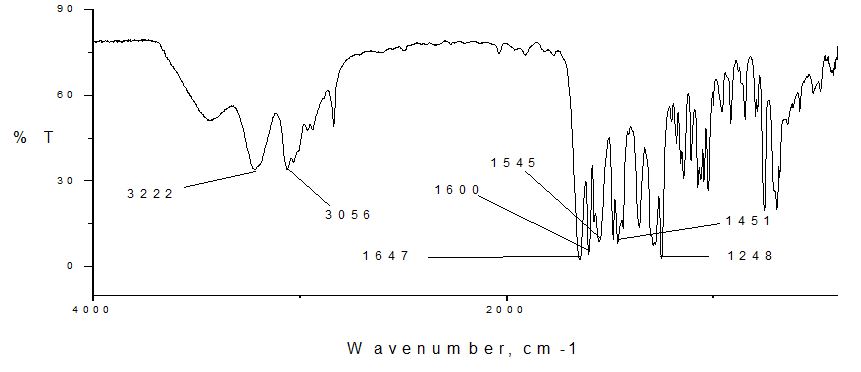


**^1^H NMR Spectrum of (E)-2-hydroxy-N'-(2-hydroxybenzylidene)benzohydrazide (9)**

**^^**

**^13^C DEPT 90 NMR Spectrum of (E)-2-hydroxy-N'-(2-hydroxybenzylidene)benzohydrazide (9)**

**^^**

**^13^C NMR Spectrum of (E)-2-hydroxy-N'-(2-hydroxybenzylidene)benzohydrazide (9)**

**IR Spectrum of (E)-2-hydroxy-N'-(2-hydroxybenzylidene)benzohydrazide (9)**

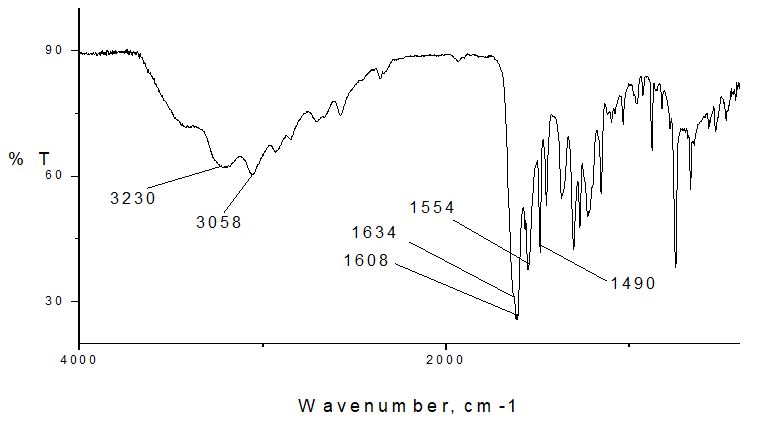


**^1^H NMR Spectrum of (E)-2-hydroxy-N'-(3-hydroxybenzylidene)benzohydrazide (10)**

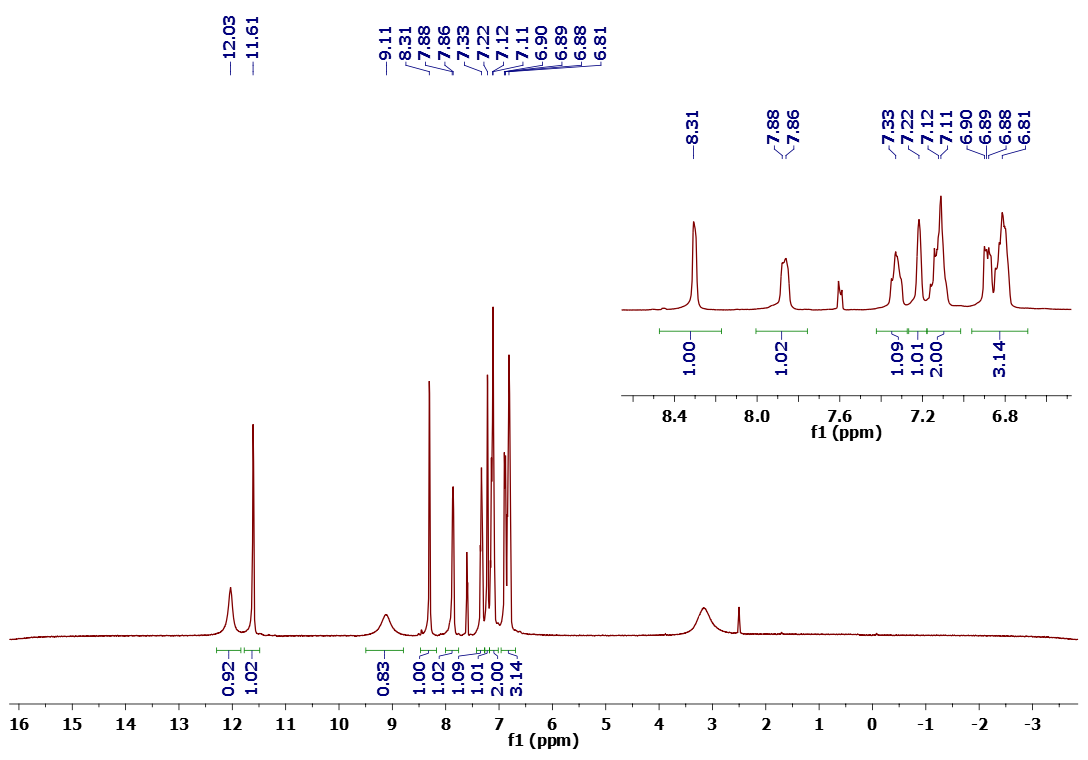


**^13^C DEPT 90 NMR Spectrum of (E)-2-hydroxy-N'-(3-hydroxybenzylidene)benzohydrazide (10)**

**^13^C NMR Spectrum of (E)-2-hydroxy-N'-(3-hydroxybenzylidene)benzohydrazide (10)**

**IR Spectrum of (E)-2-hydroxy-N'-(3-hydroxybenzylidene)benzohydrazide (10)**

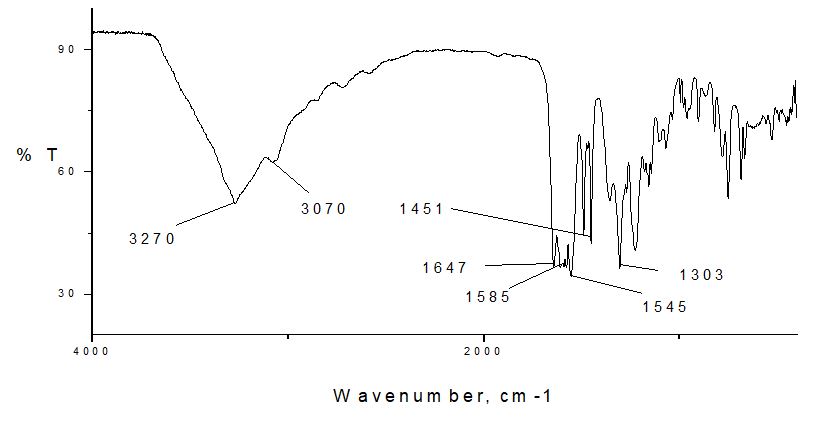


**^1^H NMR Spectrum of (E)-2-hydroxy-N'-(4-hydroxybenzylidene)benzohydrazide (11)**

**^^**

**^13^C DEPT 90 NMR Spectrum of (E)-2-hydroxy-N'-(4-hydroxybenzylidene)benzohydrazide (11)**

**^13^C NMR Spectrum of (E)-2-hydroxy-N'-(4-hydroxybenzylidene)benzohydrazide (11)**

**IR Spectrum of (E)-2-hydroxy-N'-(4-hydroxybenzylidene)benzohydrazide (11)**

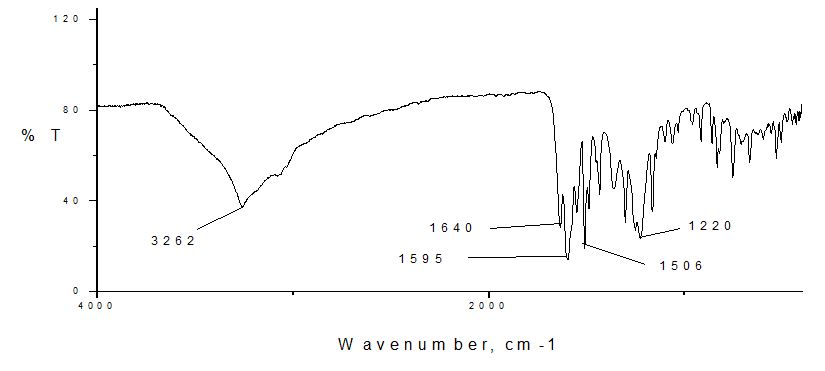


**^1^H NMR Spectrum of (E)-N'-(3,4-dihydroxybenzylidene)-2-hydroxybenzohydrazide (12)**

**^
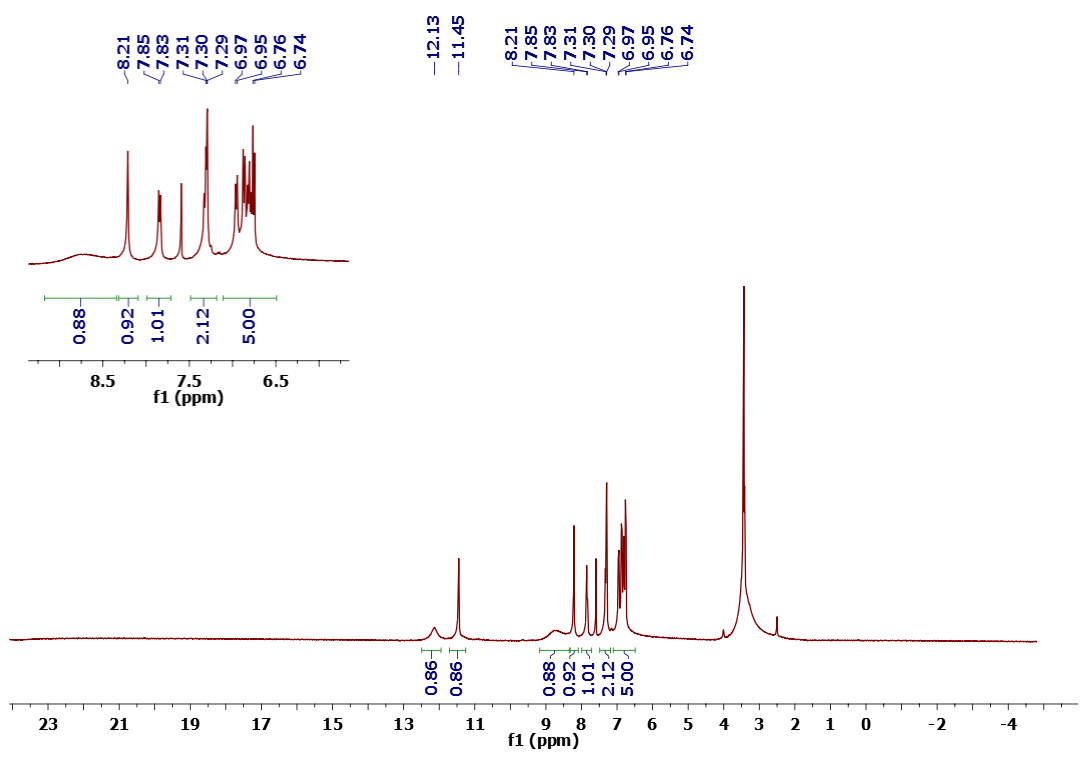
^**

**^13^C DEPT 90 NMR Spectrum of (E)-N'-(3,4-dihydroxybenzylidene)-2-hydroxybenzohydrazide (12)**

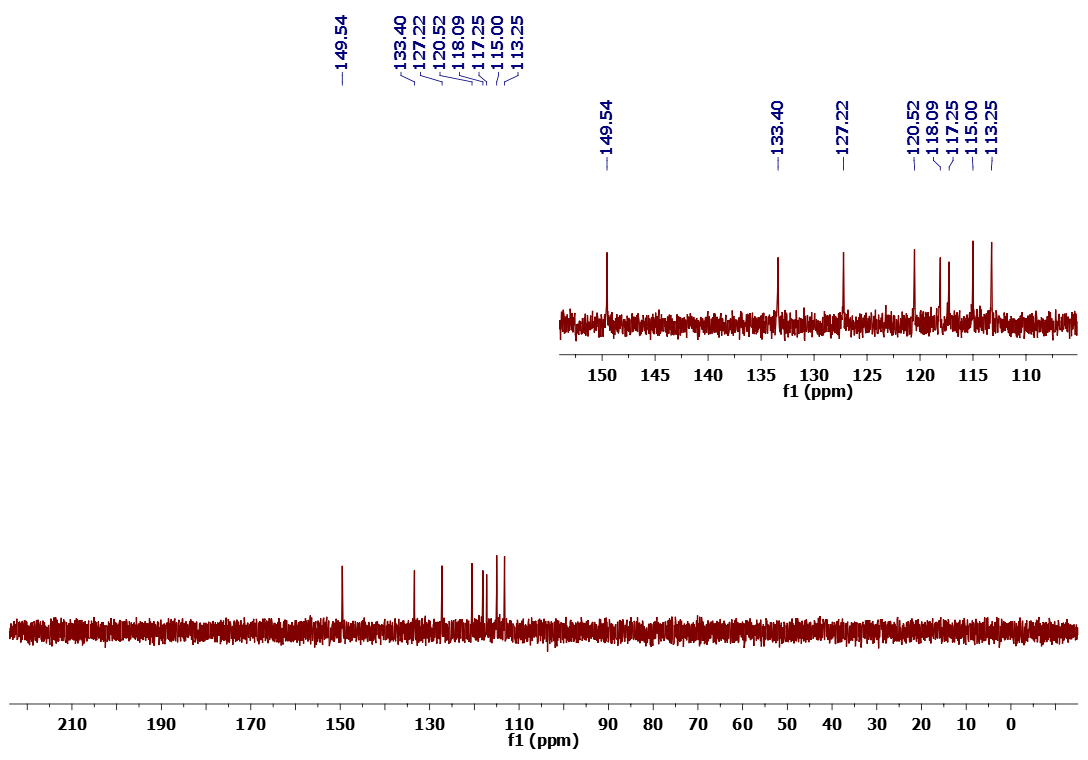


**^13^C NMR Spectrum of (E)-N'-(3,4-dihydroxybenzylidene)-2-hydroxybenzohydrazide (12)**

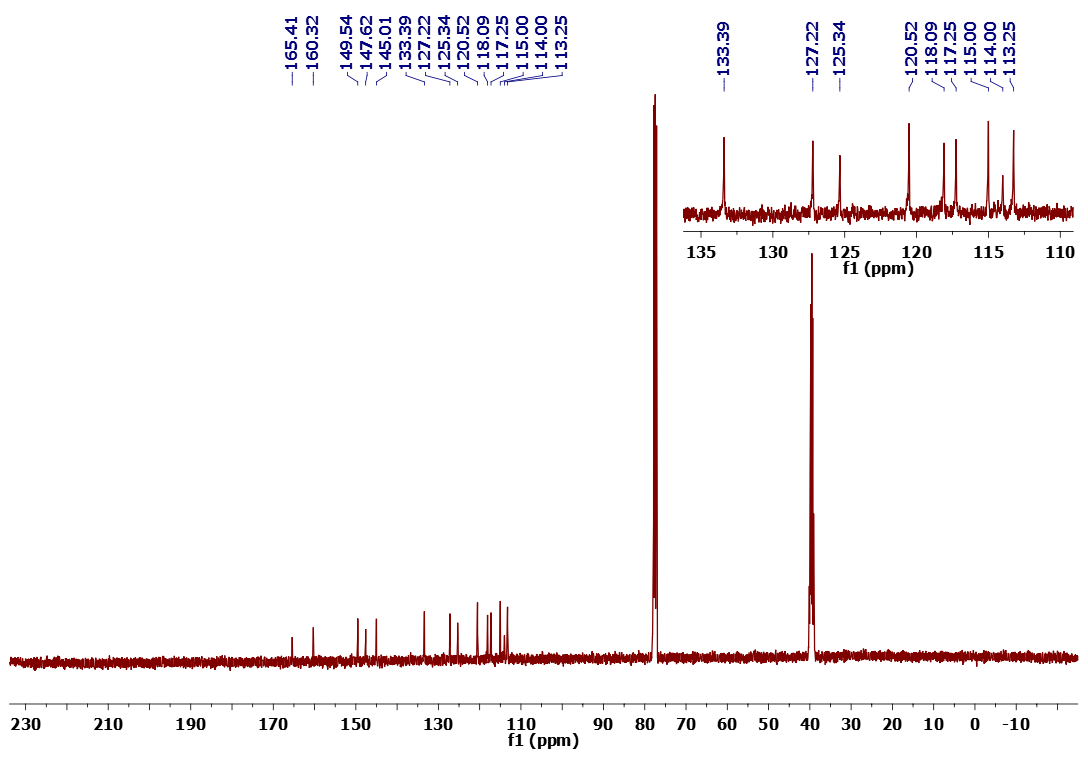


**IR Spectrum of (E)-N'-(3,4-dihydroxybenzylidene)-2-hydroxybenzohydrazide (12)**

**^
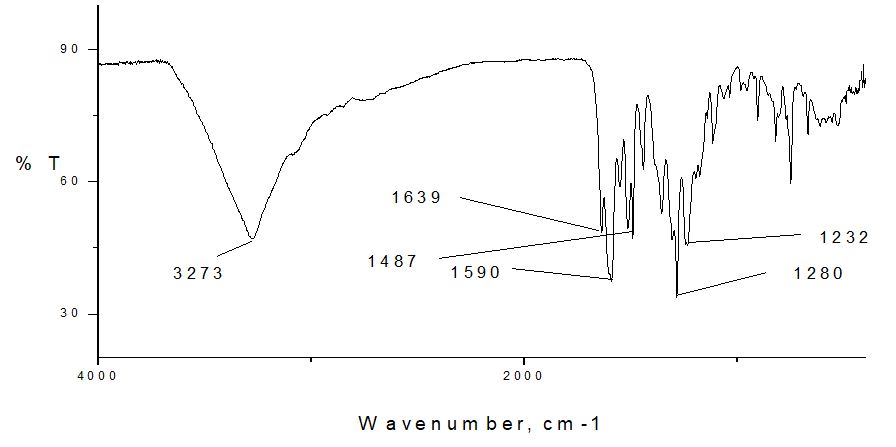
^**

**^1^H NMR Spectrum of (E)-N'-(2,4-dihydroxybenzylidene)-2-hydroxybenzohydrazide (13)**

**^
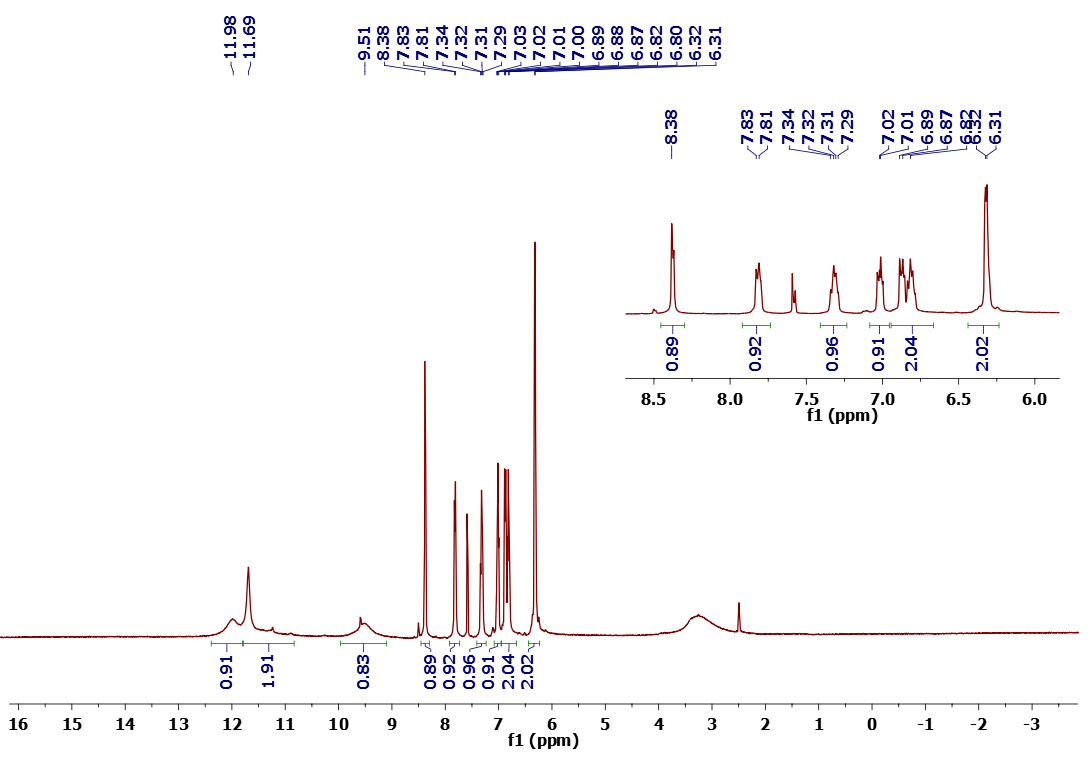
^**

**^13^C DEPT 90 NMR Spectrum of (E)-N'-(2,4-dihydroxybenzylidene)-2-hydroxybenzohydrazide (13)**

**^^**

**^13^C NMR Spectrum of (E)-N'-(2,4-dihydroxybenzylidene)-2-hydroxybenzohydrazide (13)**

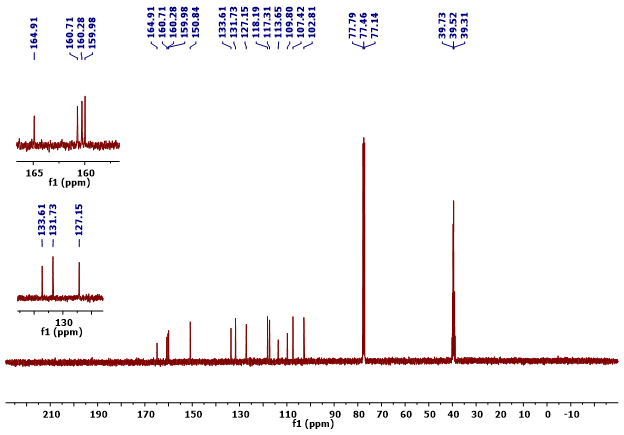


**IR Spectrum of (E)-N'-(2,4-dihydroxybenzylidene)-2-hydroxybenzohydrazide (13)**

**^
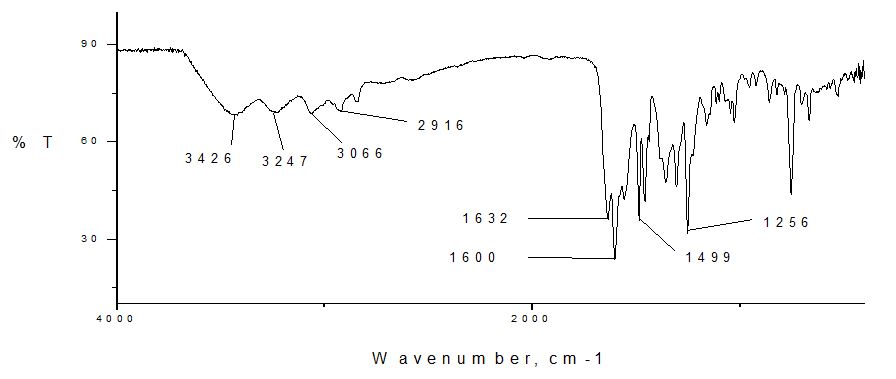
^**

**^1^H NMR Spectrum of (E)-2-hydroxy-N'-(2,3,4-trihydroxybenzylidene)benzohydrazide (14)**

**^
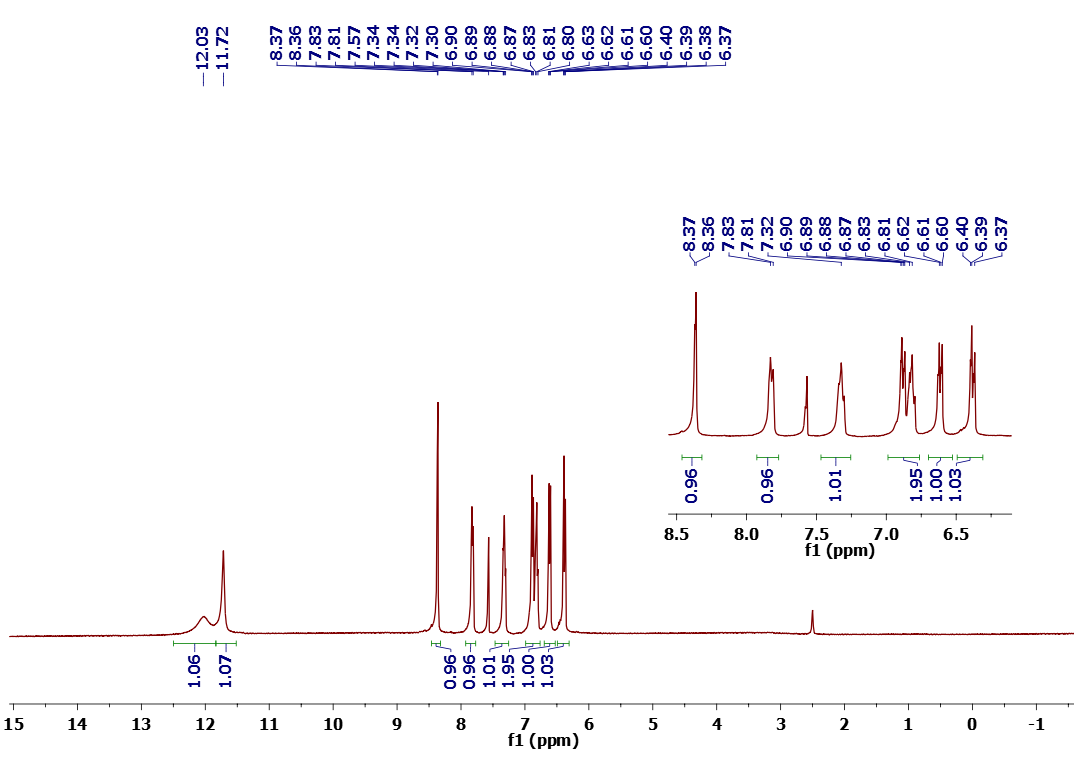
^**

**^13^C DEPT 90 NMR Spectrum of (E)-2-hydroxy-N'-(2,3,4-trihydroxybenzylidene)benzohydrazide (14)**

**^^**

**^13^C NMR Spectrum of (E)-2-hydroxy-N'-(2,3,4-trihydroxybenzylidene)benzohydrazide (14)**

**IR Spectrum of (E)-2-hydroxy-N'-(2,3,4-trihydroxybenzylidene)benzohydrazide (14)**

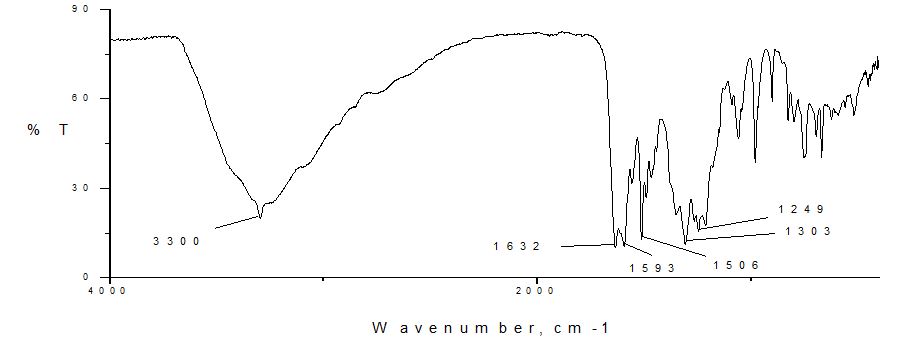


**^1^H NMR Spectrum of (E)-2-hydroxy-N'-(4-hydroxy-3-methoxybenzylidene)benzohydrazide (15)**

**^
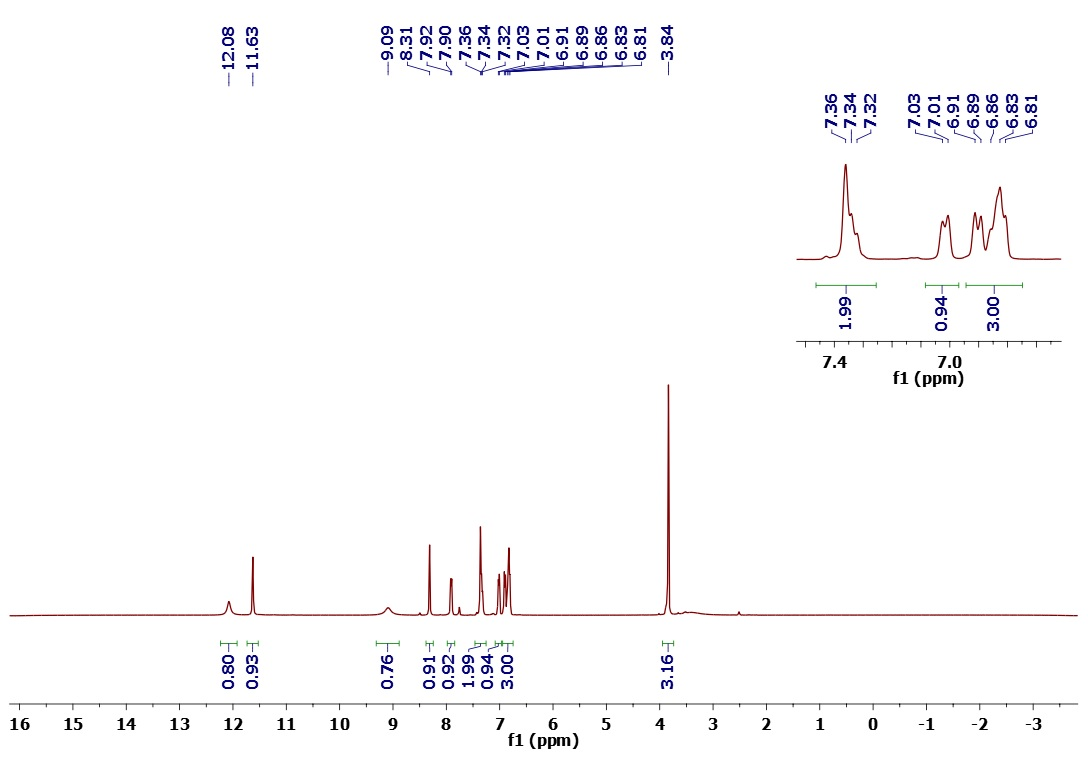
^**

**^13^C DEPT 90 NMR Spectrum of (E)-2-hydroxy-N'-(4-hydroxy-3-methoxybenzylidene)benzohydrazide (2m should be corrected to 2k)**

**^
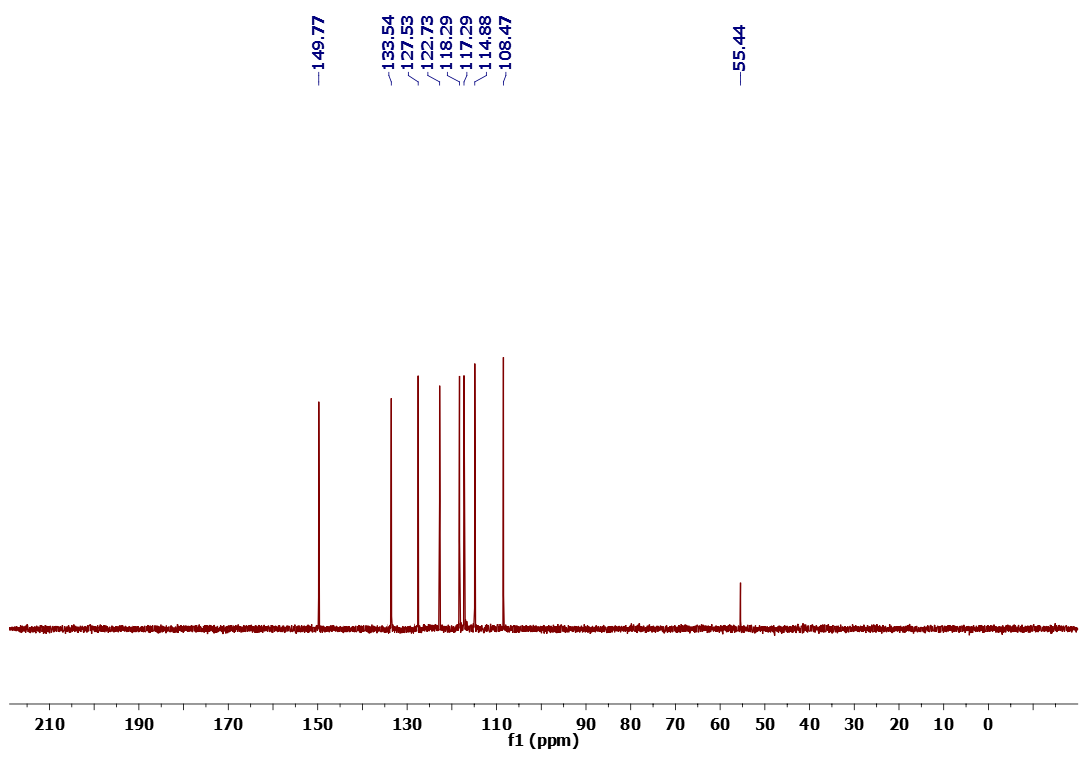
^**

**^13^C NMR Spectrum of (E)-2-hydroxy-N'-(4-hydroxy-3-methoxybenzylidene)benzohydrazide (15)**

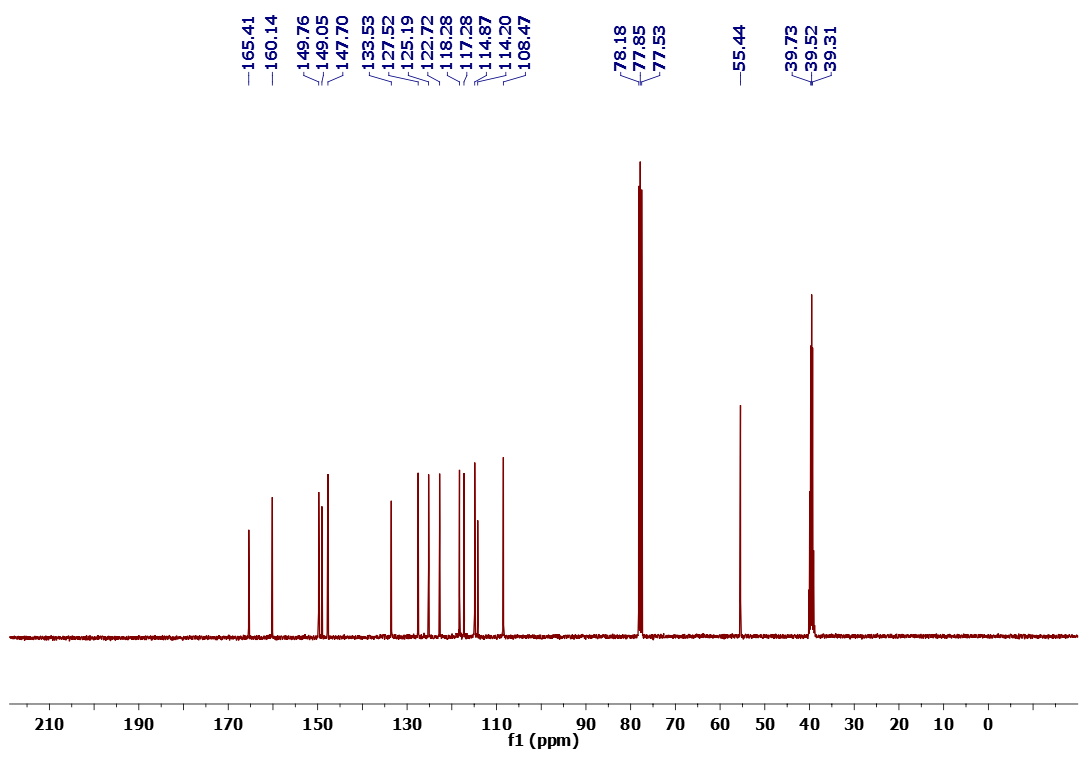


**IR Spectrum of (E)-2-hydroxy-N'-(4-hydroxy-3-methoxybenzylidene)benzohydrazide (15)**

**^
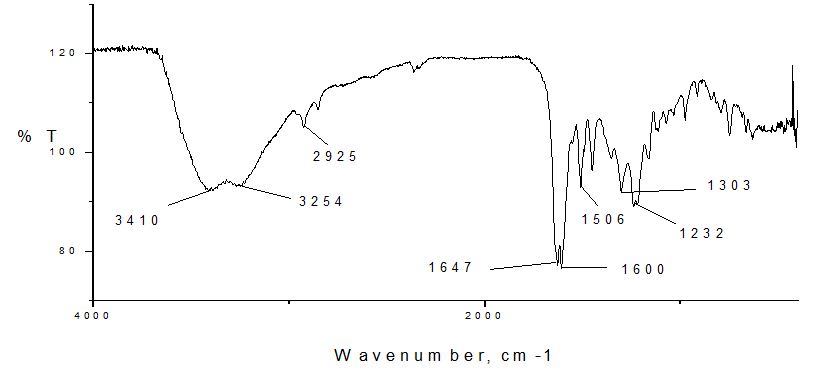
^**

**^1^H NMR Spectrum of (E)-2,4-dihydroxy-N'-(3-hydroxybenzylidene)benzohydrazide (16)**

**^
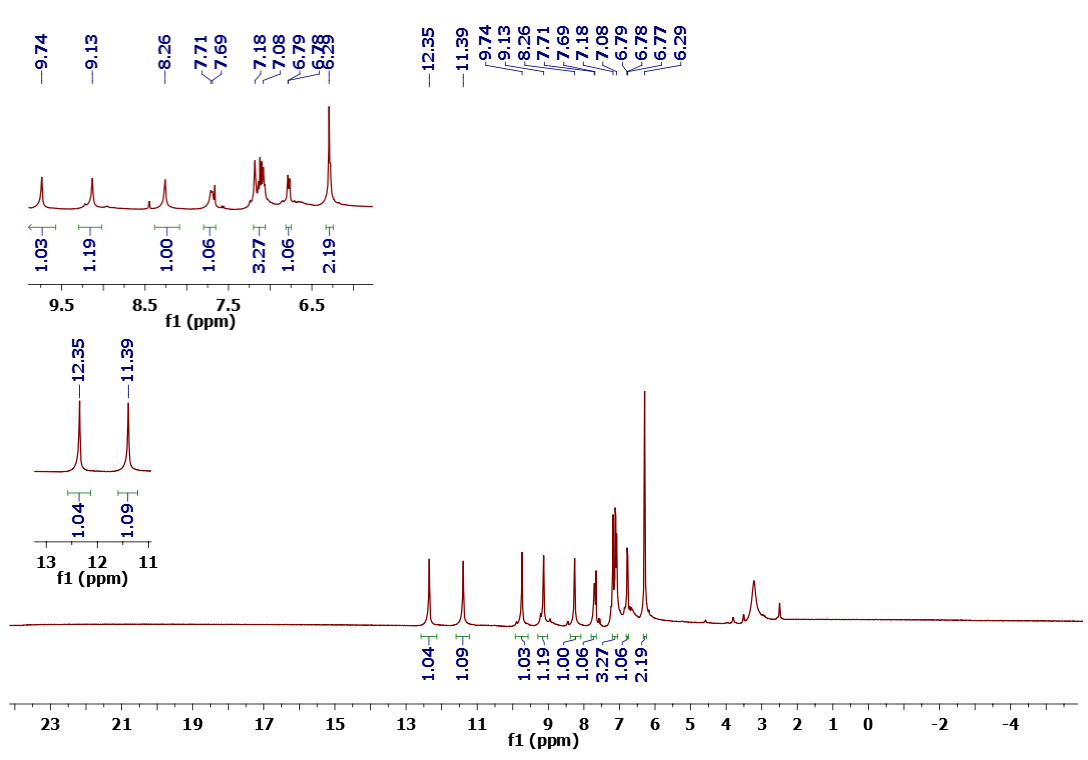
^**

**^13^C DEPT 90 NMR Spectrum of (E)-2,4-dihydroxy-N'-(3-hydroxybenzylidene)benzohydrazide (16)**

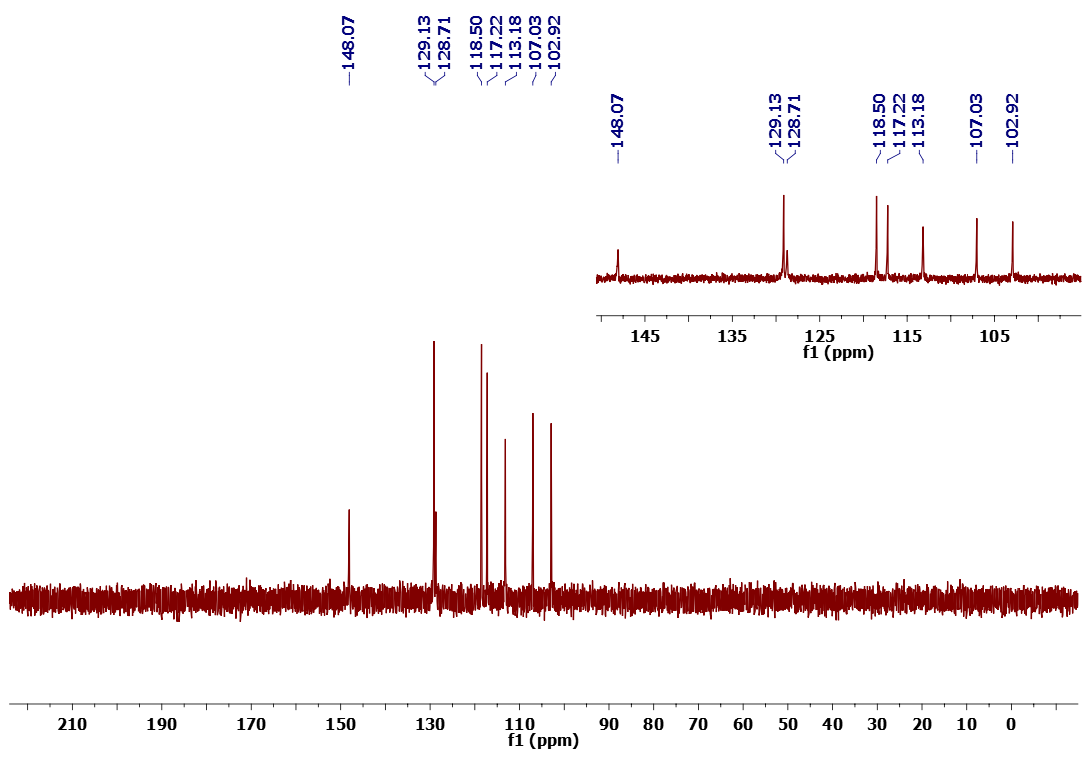


**^13^C NMR Spectrum of (E)-2,4-dihydroxy-N'-(3-hydroxybenzylidene)benzohydrazide (16)**

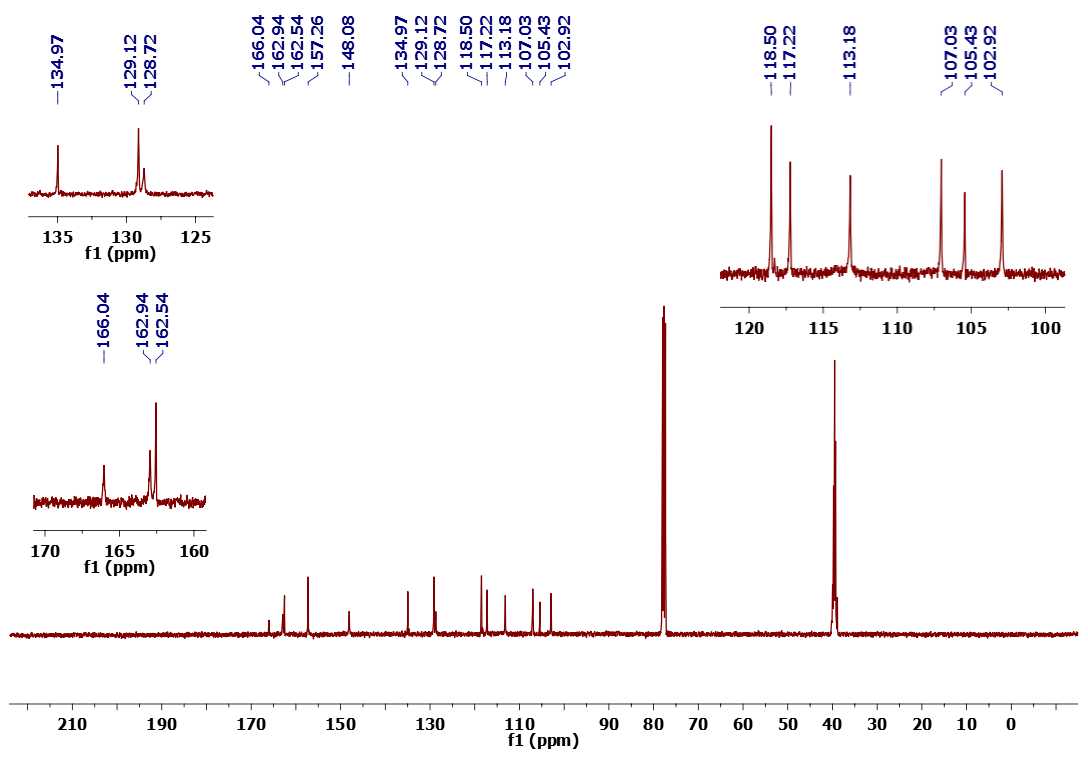


**IR Spectrum of (E)-2,4-dihydroxy-N'-(3-hydroxybenzylidene)benzohydrazide (16)**

**^
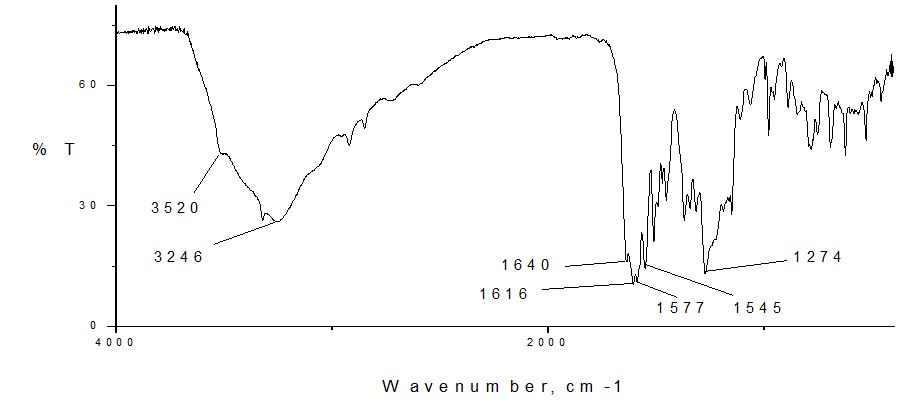
^**

**^1^H NMR Spectrum of (E)-2,4-dihydroxy-N'-(4-hydroxybenzylidene)benzohydrazide (17)**

**^
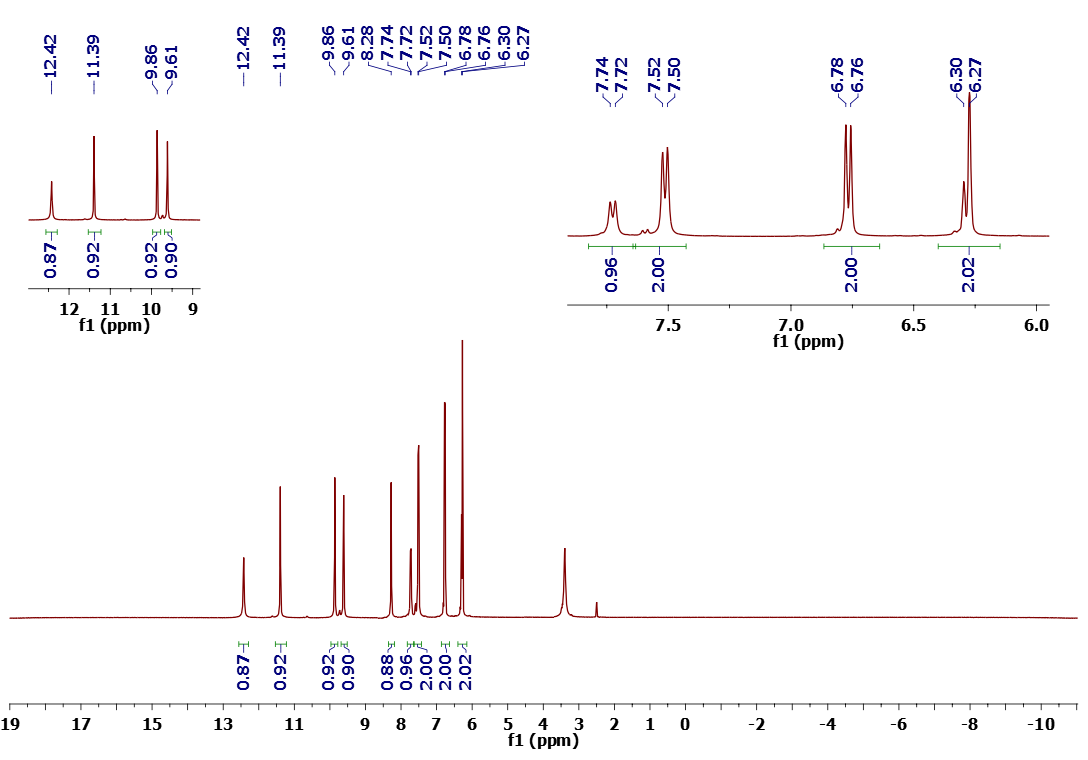
^**

**^13^C DEPT 90 NMR Spectrum of (E)-2,4-dihydroxy-N'-(4-hydroxybenzylidene)benzohydrazide (17)**

**^
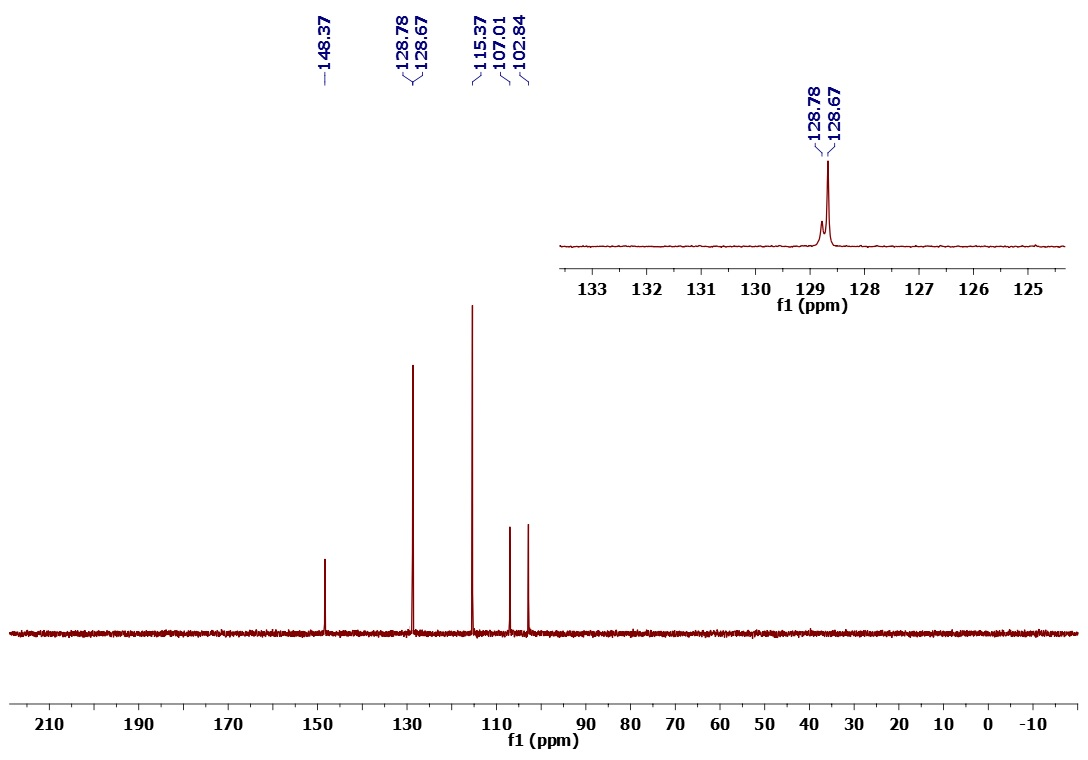
^**

**^13^C NMR Spectrum of (E)-2,4-dihydroxy-N'-(4-hydroxybenzylidene)benzohydrazide (17)**

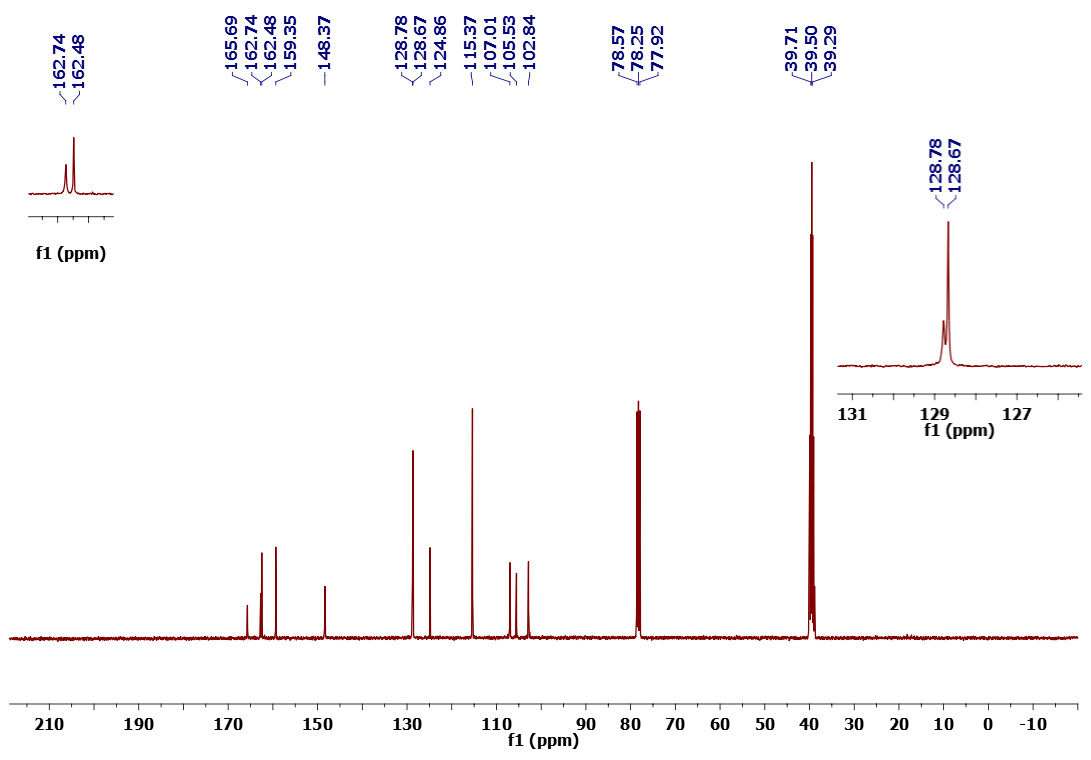


**IR Spectrum of (E)-2,4-dihydroxy-N'-(4-hydroxybenzylidene)benzohydrazide (17)**

**^
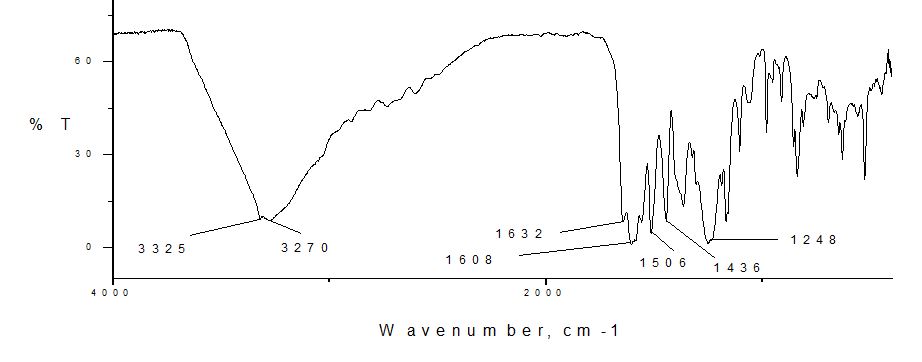
^**

**^1^H NMR Spectrum of (E)-N'-(3,4-dihydroxybenzylidene)-2,4-dihydroxybenzohydrazide (18)**

**^
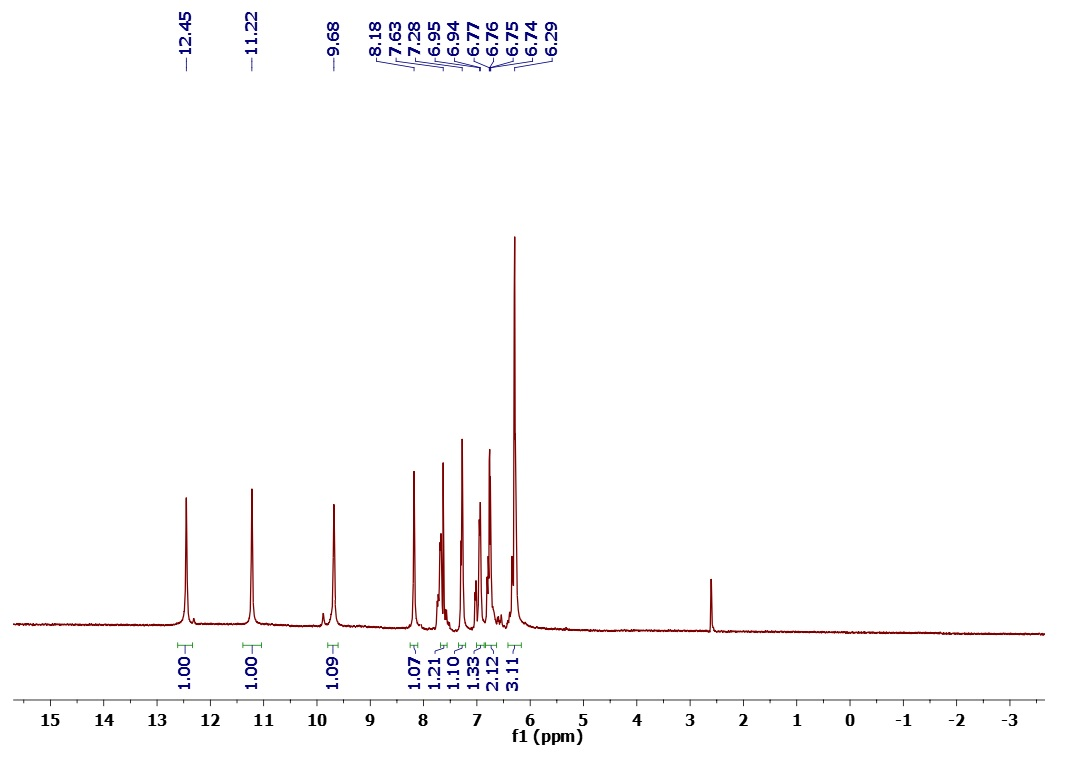
^**

**^13^C DEPT 90 NMR Spectrum of (E)-N'-(3,4-dihydroxybenzylidene)-2,4-dihydroxybenzohydrazide (18)**

**^
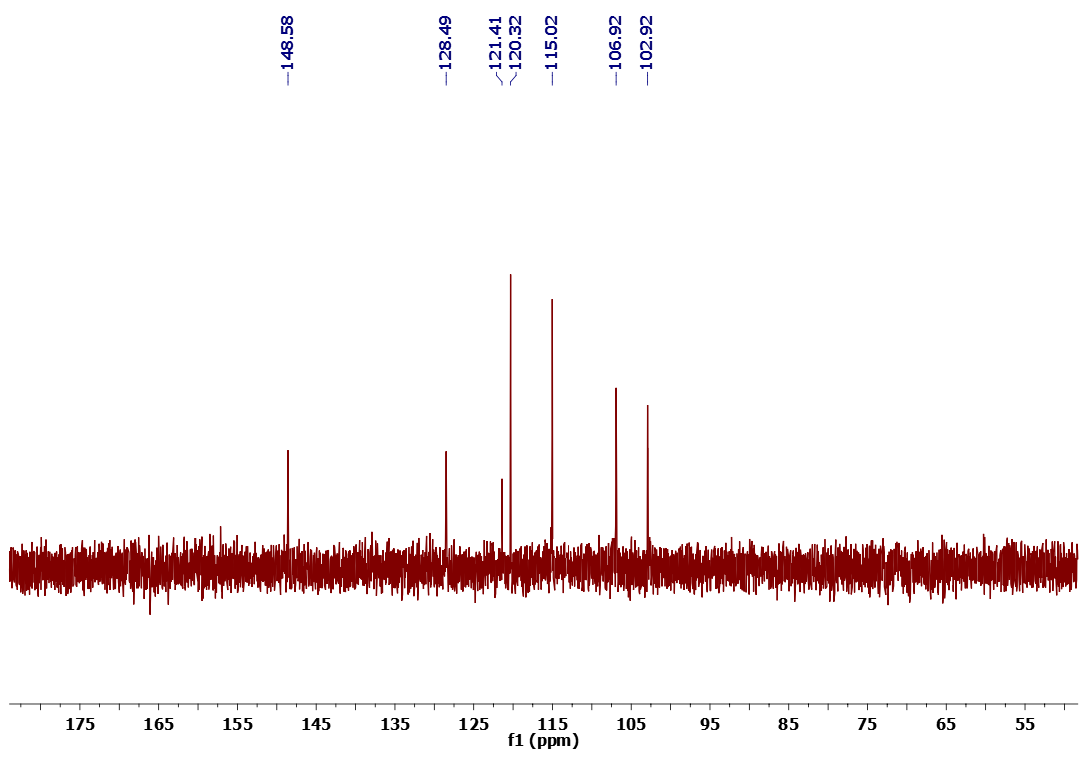
^**

**^13^C NMR Spectrum of (E)-N'-(3,4-dihydroxybenzylidene)-2,4-dihydroxybenzohydrazide (18)**


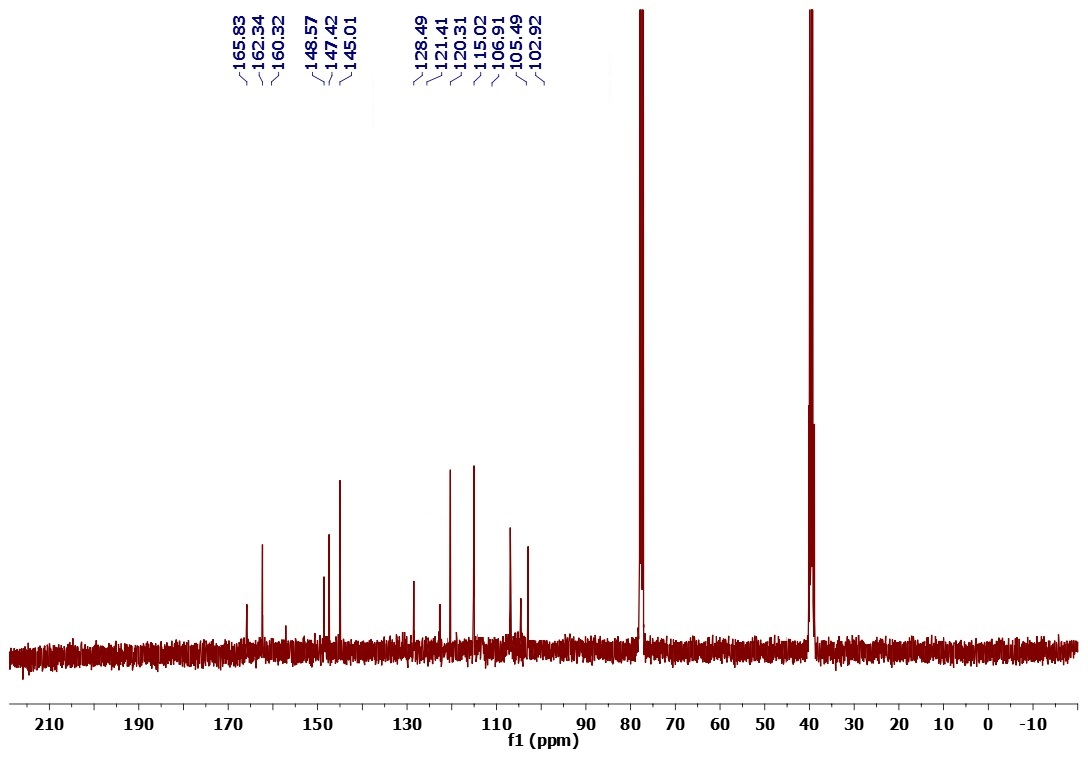


**IR Spectrum of (E)-N'-(3,4-dihydroxybenzylidene)-2,4-dihydroxybenzohydrazide (18)**

**^
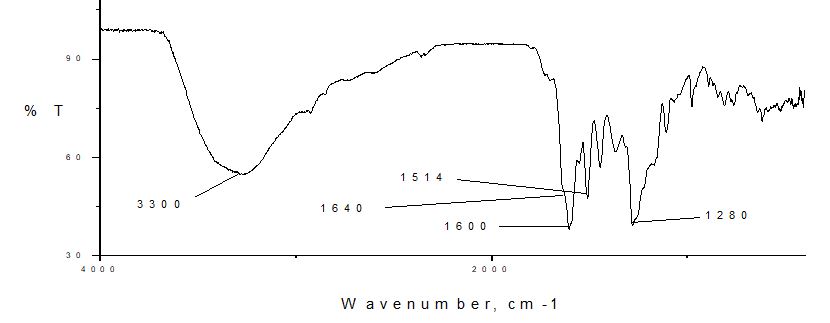
^**

**^1^H NMR Spectrum of (E)-N'-(2,4-dihydroxybenzylidene)-2,4-dihydroxybenzohydrazide (19)**


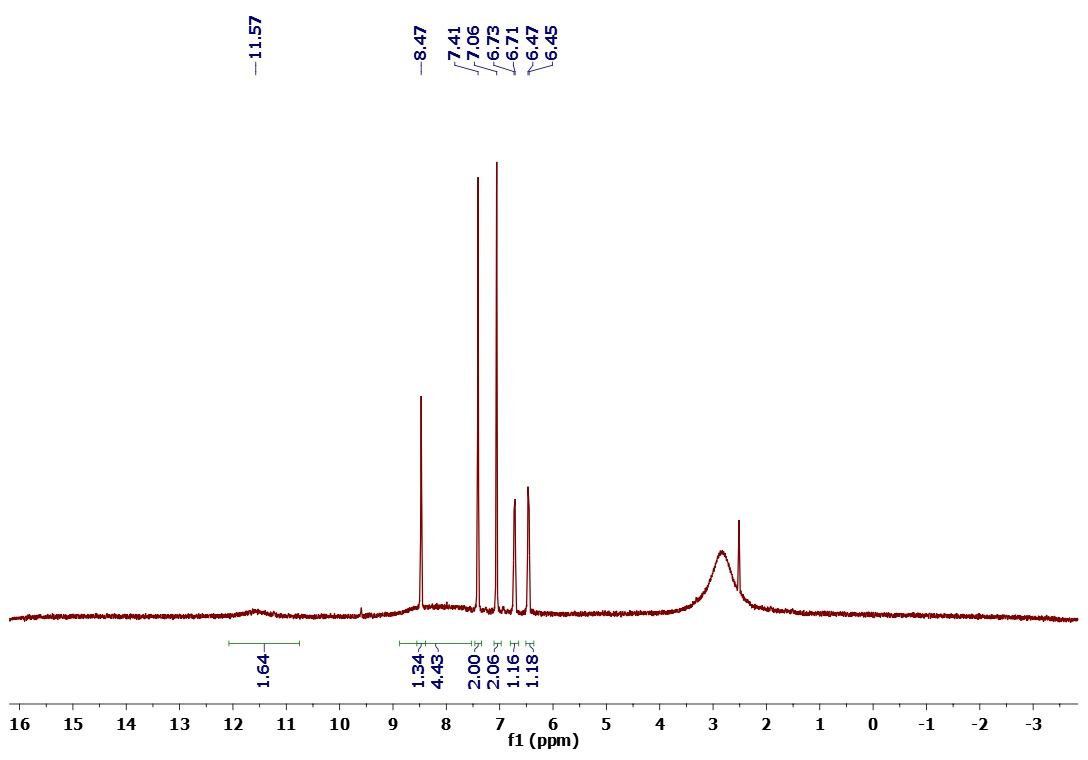


**^13^C NMR Spectrum of** **(E)-N'-(2,4-dihydroxybenzylidene)-2,4-dihydroxybenzohydrazide (19)**


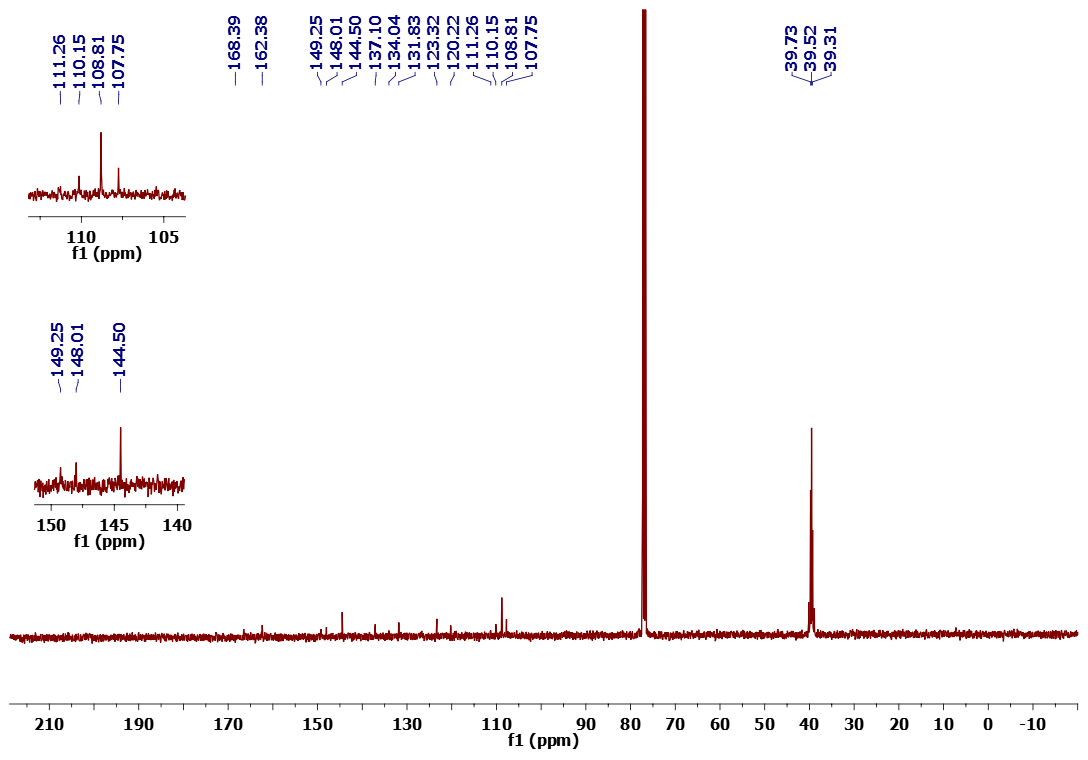


**IR Spectrum of (E)-N'-(2,4-dihydroxybenzylidene)-2,4-dihydroxybenzohydrazide (19)**


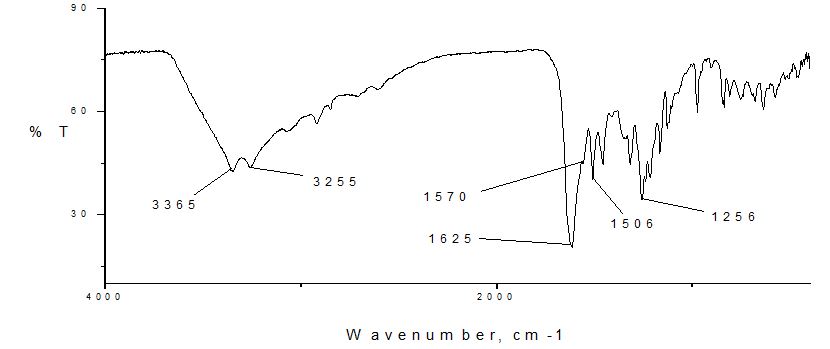


**^1^H NMR Spectrum** **of (E)-3,4,5-trihydroxy-N'-(3-hydroxybenzylidene)benzohydrazide (20)**

**^
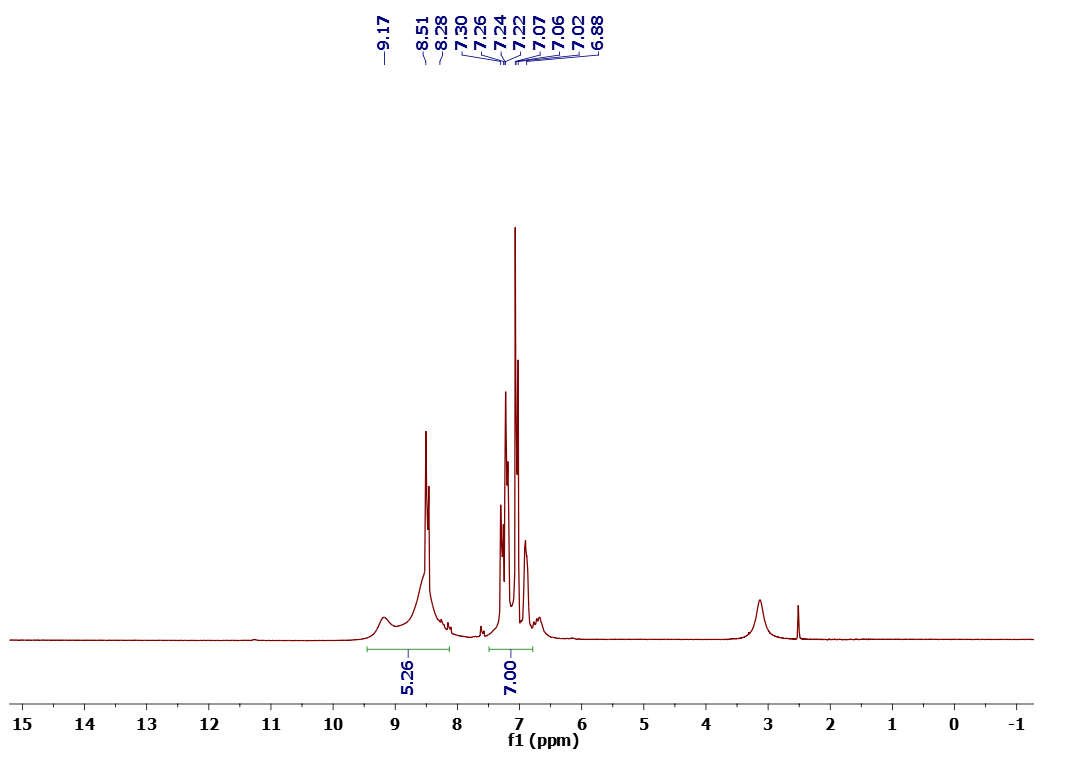
^**

**^13^C DEPT 90 NMR Spectrum of (E)-3,4,5-trihydroxy-N'-(3-hydroxybenzylidene)benzohydrazide (20)**

**^
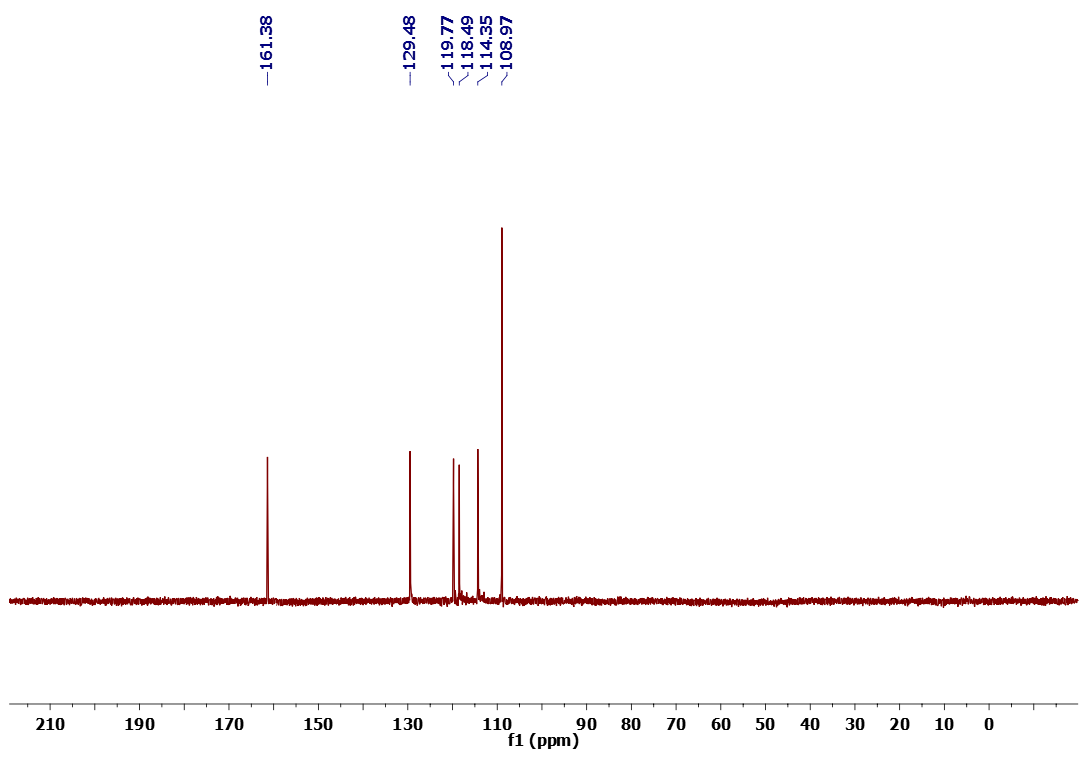
^**

**^13^C NMR Spectrum of (E)-3,4,5-trihydroxy-N'-(3-hydroxybenzylidene)benzohydrazide (20)**


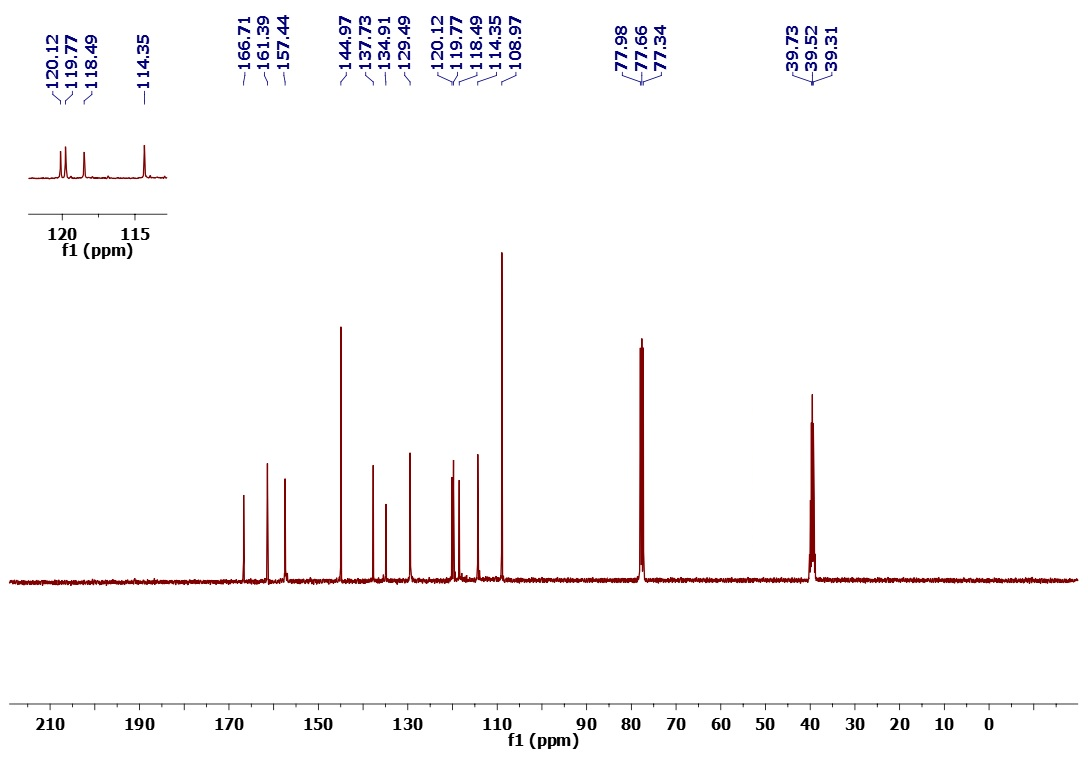


**IR Spectrum of (E)-3,4,5-trihydroxy-N'-(3-hydroxybenzylidene)benzohydrazide (20)**

**^
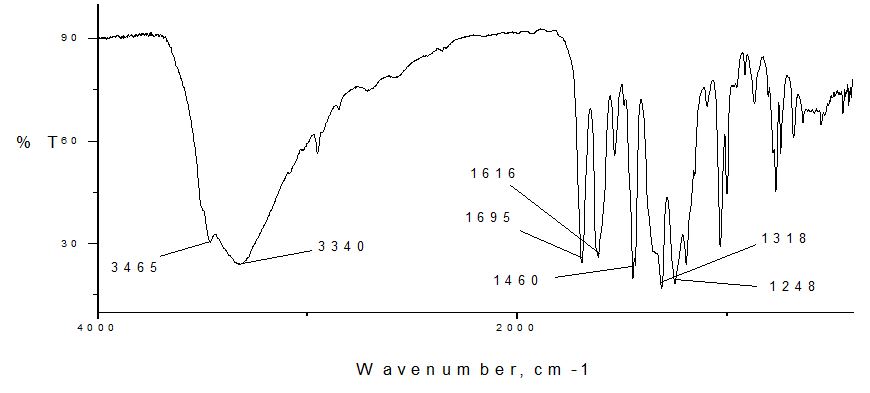
^**
